# Synthesis, Spectroscopy, and Computational Analysis of Photoluminescent Bis(aminophenyl)-Substituted Thiophene Derivatives

**DOI:** 10.1002/cphc.201201006

**Published:** 2013-02-26

**Authors:** Daniel Lumpi, Ernst Horkel, Felix Plasser, Hans Lischka, Johannes Fröhlich

**Affiliations:** [a] Institute of Applied Synthetic Chemistry, Vienna University of TechnologyGetreidemarkt 9, 1060 Vienna (Austria), Fax: (+43) 1-58801-15499 E-mail: ernst.horkel@tuwien.ac.at; [b] Institute for Theoretical Chemistry, University of ViennaWähringerstrasse 17, 1090 Vienna (Austria); [c] Department of Chemistry and Biochemistry, Texas Tech UniversityLubbock, Texas 79409-1061 (USA)

**Keywords:** density functional calculations, fluorescence spectroscopy, organic electronics, vibrational broadening, Wigner distribution

## Abstract

Substituted oligothiophenes have a long history in the field of organic electronics, as they often combine outstanding electro-optical properties with the ease of synthesis. To assist the rational selection of the most promising structures to be synthesized, there is the demand for tools that allow prediction of the properties of the materials. In this study, we present strategies for synthesis and computational characterization, with respect to the fluorescence behavior of oligothiophene-based materials for organoelectronic applications. In a combined approach, sophisticated computational methodologies are directly compared to experimental results. The M06-2X functional in combination with the polarizable continuum model in a state-specific formulation for excited-state solvation proved to be particularly reliable. In addition, a semiclassical approach for describing the vibrational broadening of the spectra is employed. As a result, a robust procedure for the prediction of the fluorescence spectra of oligothiophene derivatives is presented.

## 1. Introduction

Organic electronics have gained much interest from industry in recent years, and this field covers the use of organic semiconductors within electronic devices, for example, OFETs (organic field-effect transistors), OLEDs (organic light-emitting diodes), and OPVs (organic photovoltaics).^1^ The application of these “plastic electronics” in the field of display technology enables the construction of very-thin, flexible, energy-efficient, light-weight panels that are highly suitable for customer electronic displays.^2^ Considering the benefits of this technology, very intense academic and industrial research activities are on the way to overcome deficits of these materials (e.g. limited lifetimes). Thus, the quest for novel materials is an ongoing process, especially because OLEDs are believed to have enormous economic potential in the near future.

**Figure 1:**
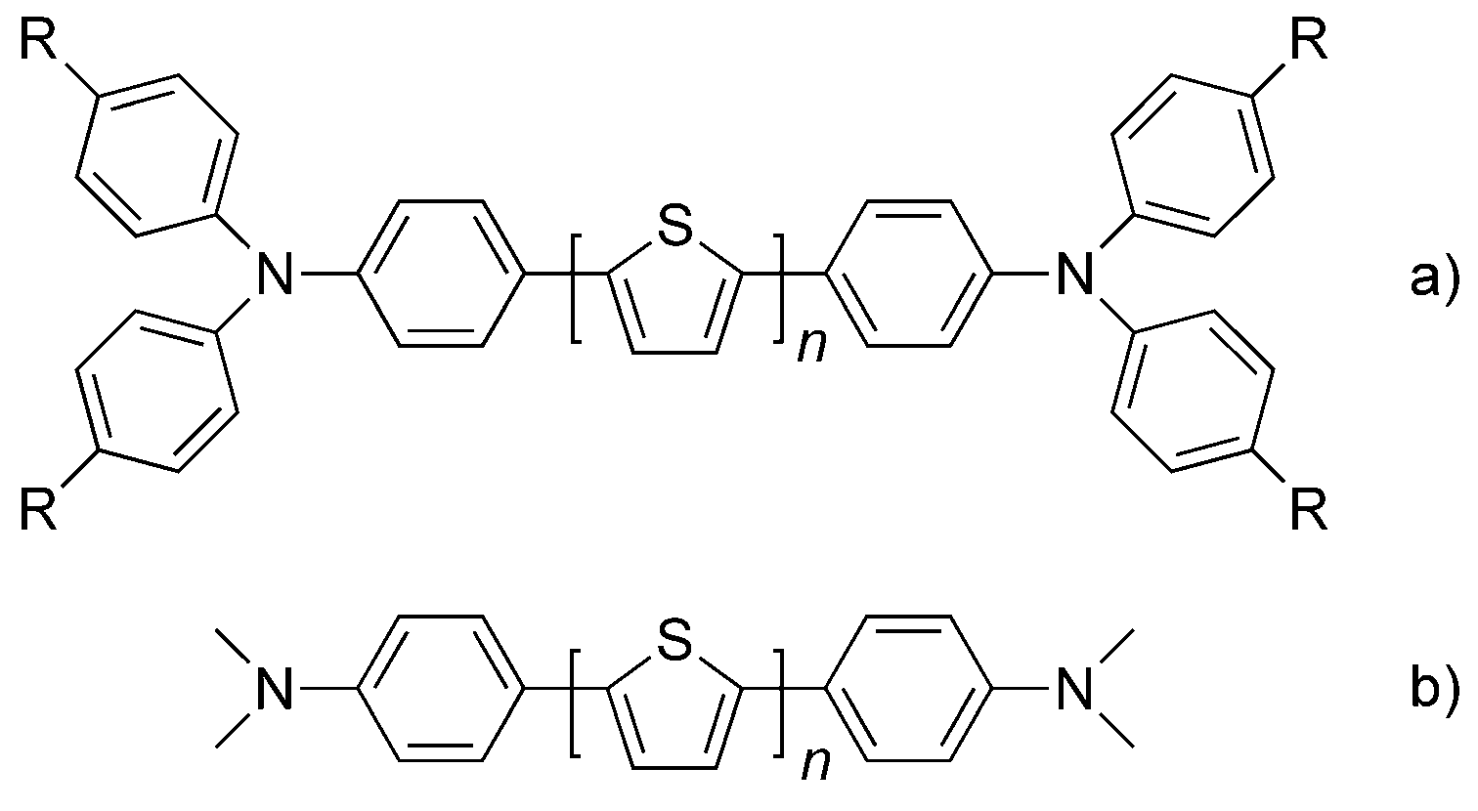
a) Substance class BRA-*n*T (BMA-*n*T: R=CH_3_, *n*=1–4; studied by Noda et al.). b) Model compounds DMA-*n*T (*n*=1–3).

However, the synthesis of novel organic compounds can be an expensive procedure, considering both the costs of fine chemicals, reagents, and catalysts and man power. Thus, it would be a great benefit to obtain information on the physical properties of interest of the substance prior to synthesis. On the basis of such knowledge, the most promising candidates can be selected within a substance class under investigation. In the case of OLED materials, luminescent properties are of particular interest. As the molecules used are, in general, of considerable size, the availability of reliable and computationally efficient methods for the prediction of photoluminescent properties is essential. Poly- and oligothiophene-based compounds are widely used within the field of organic electronics.^3^ They display remarkable conductivity with excellent chemo- and thermostability, and they are, in general, readily synthesized or structurally modified to meet electronic and spectroscopic requirements. Hence, one of our ongoing research topics involves the modification of the substance class BRA-*n*T (Figure 1).

A small subset, namely, BMA-*n*T, was studied by Noda et al.^4^ and found to be suitable for the fabrication of single- and multilayer OLEDs. Moreover, it was shown that the use of triarylamine subunits had a positive influence on both the conductivity and the tendency to form amorphous glasses. Thus, combinations of (oligo)thiophenes with triaryl moieties are widely used in all fields of organic electronics.^5^

For BRA-*n*T, variation of both the conjugation length *n* of the oligothiophene linker as well as the R substituents of the triarylamine are interesting, especially with regard to the effects of the applied modifications on the electrooptical characteristics. Emission wavelengths were calculated by time-dependent density functional theory (TDDFT),^6^ as recent developments in this area allow the reliable and efficient treatment of molecules of considerable size, which makes it the method of choice for this study. Solvent effects were taken into account by applying a polarizable continuum model (PCM). Aside from its standard linear response formalism for excited states, the state-specific solvation model was also used.^7^ Moreover, to understand and describe spectral broadening, which is an important factor in, for example, the design of white OLEDs, we included vibrational information. Simulations based on the Wigner distribution of the vibrational ground state of the first excited electronic state proved to be appropriate to perform this task. As a result of this project, a reliable procedure can be presented for the calculation of the entire fluorescence spectra of oligothiophene-based compounds.

## 2. Results and Discussion

**Scheme 1:**
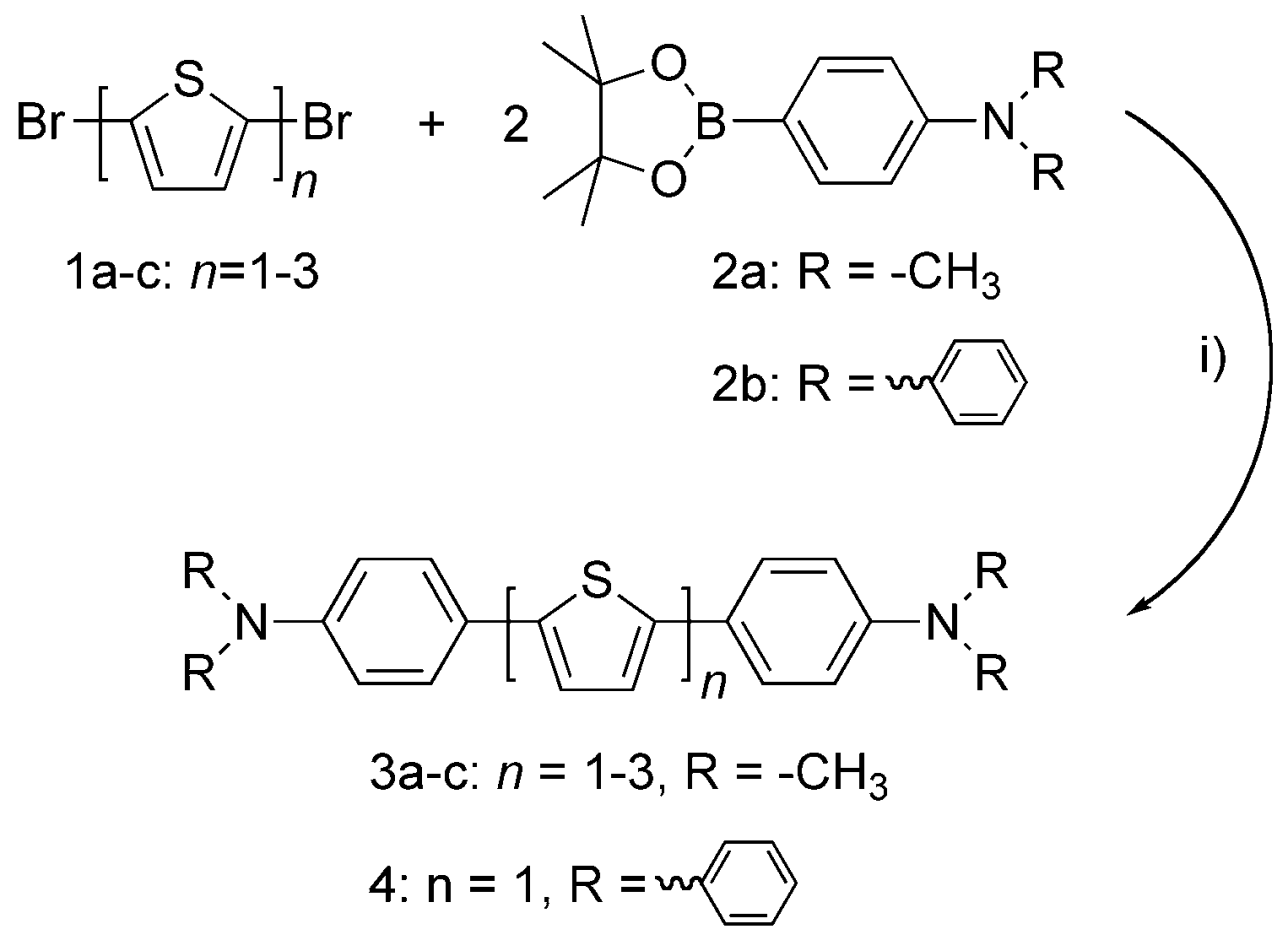
Synthesis of **3 a**–**c** (DMA-*n*T) and **4** (BHA-1T). Reagents and conditions: i) **1 a**–**c** (1.0 mmol), boronic ester **2 a**,**b** (3.0 mmol), degassed *i*PrOH/H_2_O, KO*t*Bu (3.0 mmol), (IPr)Pd(allyl)Cl (2.0 mol % for **3 a**–**c**, 5.0 mol % for **4**), reflux (≍80 °C), 1–2 h.

As already mentioned, bis-substituted derivatives containing (oligo)thiophene substructures have gained considerable attention in the field of organic functional materials [e.g. BHA-1T^8^ (**4**); R=phenyl, *n*=1; Scheme 1]. As a consequence, a large number of oligothiophene derivatives have been synthesized and characterized. Additionally, these types of compounds have been subjected to theoretical studies. These cover, for example, investigations of vibrational (IR and Raman)^9^ and electrochemical^10^ properties as well as the treatment of excited states.^11^ However, in most cases no experimental data was supplied for comparison with the calculated properties. In contrast, our study focuses on the application of modern and computationally affordable methods to molecules relevant in the field of materials science, as we combine our theoretical results with experimental data of well-characterized compounds. As a future perspective, these data can be interesting for subsequent investigations, for example, to provide reference data for other approaches. To minimize the computational effort during the initial stage of the project, simplifications were applied to BRA-*n*T. Replacement of the relatively large triarylamine system with a considerably smaller dimethyl(4-aminophenyl) group leads to model compounds **3 a**–**c** (DMA-*n*T, *n*=1–3; Scheme 1), which were used as mimics to benchmark the applied computational techniques and parameters used within the calculations. Compound DMA-1T was previously characterized in 1998 when obtained as a byproduct during the synthesis of 1,2-dithiin derivatives.^12^ However, a reliable synthetic protocol has not been published so far. A selective approach toward DMA-*n*T and BHA-1T could be developed through Suzuki cross-coupling strategies by utilizing dibromothiophenes **1 a**–**c** and dioxaborolanes **2 a**, **b** (Scheme 1).

**Figure 2:**
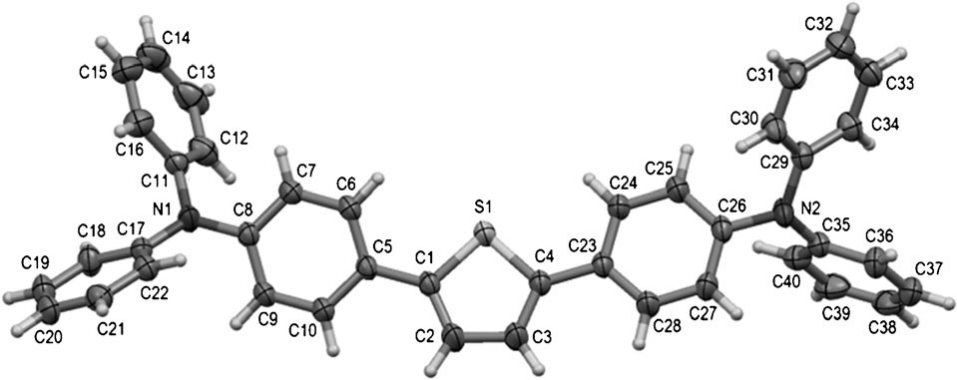
Molecular structure of **4** (BHA-1T).

The best results were achieved by using the N-heterocyclic carbene (NHC) catalyst (IPr)Pd(allyl)Cl,^13^ which afforded the desired materials in good to excellent yields. According to our experimental experience, this catalytic system, in general, shows high reactivity for the benzene–thiophene linkage. Crystallization of BHA-1T from cyclohexane yielded single-crystalline samples; the molecular structure of **4** is illustrated in Figure 2.

Relative to other excited-state methods, the TDDFT approach is favorable because of its low scaling behavior with respect to increasing size of the system, which leads to acceptable computational costs, even for large systems. However, the results obtained can strongly depend on the functional used.^14^ Standard functionals may particularly suffer from overstabilization of charge-transfer excited states^15^ and are not reliable for large conjugated systems.^16^ To select an appropriate functional for the description of the fluorescence properties of the substance class under investigation, three different functionals (i.e. B3LYP,^17^ PBE0,6a, 18 M06-2X6b) were considered. With these functionals, benchmark calculations were performed on the model DMA-*n*T compounds and solution-based computational results were compared to the experimental values.

**Figure 3:**
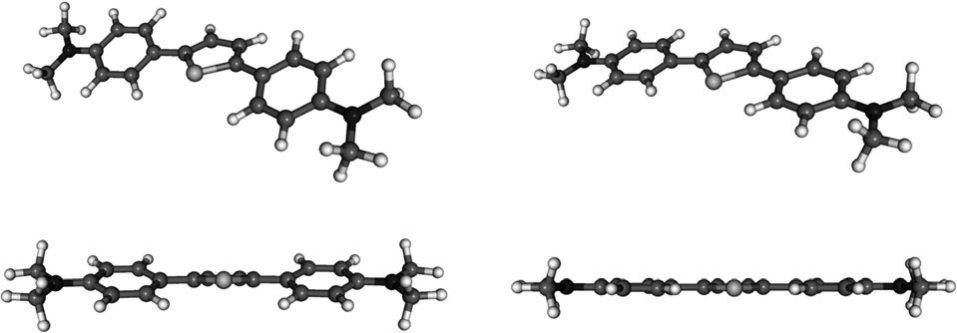
Optimized geometries of DMA-1T (**3 a**) (M06-2X/SVP, gas phase); left: S_0_, right: S_1_.

In Figure 3, the optimized geometries of both the ground state (S_0_) and the first excited state (S_1_) of DMA-1T are presented. A significant structural change is observed, and the most obvious difference is the torsion angle between the phenyl substituent and the linking thiophene unit. Taking the M06-2X-optimized structures as an example, this angle is about 24° in S_0_, whereas it decreases to 0° in S_1_, which thus leads to a planar excited-state geometry. By comparing the optimized geometries of S_0_ and S_1_, the same planarization can be observed for DMA-2T and DMA-3T (for illustrations see the Supporting Information, Figures S1–S3). When analyzing the bond length alternation in DMA-1T, one can recognize a significant contraction of the C1–C1′ thiophene bond as well as the C2–C3 linkage, whereas the C1–C2 bond is elongated. Moreover, the quinoidal distortion of the benzene rings is enhanced. Both phenomena, the planarization and the quinoidal distortion, are processes that are well known for systems of this type.16b, 19 Detailed information on the bond lengths in both the S_0_ and S_1_ geometries of DMA-1T is presented in Table 1.

**Table 1:** Bond lengths of DMA-1T (M06-2X/SVP, gas phase)

Bond	Length [Å]		
	S_0_	S_1_	
C1–C1′	1.4222	1.3788	
C1–C2	1.3747	1.4234	
C2–S	1.7356	1.7640	
C2–C3	1.4678	1.4162	
C3–C4	1.4018	1.4270	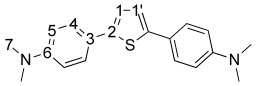
C4–C5	1.3877	1.3786	
C5–C6	1.4123	1.4192	
C6–N	1.3767	1.3738	
C7–N	1.4418	1.4424	

**Figure 4:**
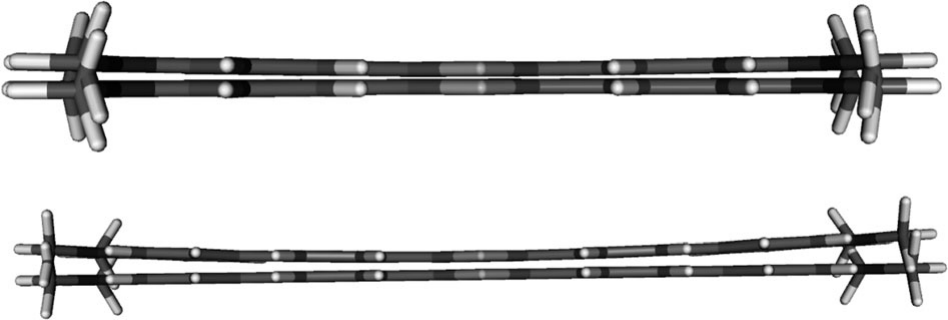
Optimized S_1_ geometries of DMA-1T (**3 a**, top) and DMA-3T (**3 c**, bottom); in both graphs: upper (bent) structure calculated with M06-2X/SVP, flat structure with B3LYP.

In a first step, gas-phase calculations for the vertical emission from the minimum geometry of the first excited state (S_1_) were performed (Table 2). First, it may be noticed that by increasing the number of repetitive thiophene units (i.e. going from DMA-1T to DMA-3T) and, therefore, elongating the π electron system, the fluorescence wavelength is also increased. By examining the results for the vertical emissions obtained by the three different functionals under investigation, decreasing wavelengths are found moving from B3LYP to PBE0 to M06-2X, which is similar to the trend reported in ref. 20. For the smallest system (i.e. DMA-1T), this difference between the excitation energies obtained from B3LYP and M06-2X calculations amounts to a shift of 33 nm (0.23 eV). In the case of DMA-3T, this difference is larger at 69 nm (0.31 eV). A comparison with the RI-CC2/TZVPP reference (Table 2) and the experimental results (see below) clearly favors the M06-2X results. When comparing the excited-state geometries obtained from the three different functionals, a nearly ideal flat structure is derived from B3LYP and PBE0 computations, whereas M06-2X leads to a slightly curved geometry (Figure 4).

**Table 2:** Calculated gas-phase vertical emissions at various levels of theory. All data denoted in nm

Level of theory	Vertical emission			
	DMA-1T	DMA-2T	DMA-3T	BHA-1T
B3LYP/SVP (6-311++G**)	440(461)	508(533)	561(590)	–
PBE0/SVP (6-311++G**)	428(446)	490(513)	539(564)	–
M06-2X/SVP (6-311++G**)	407(426)	457(480)	492(516)	431(450)
RI-CC2/TZVPP (QZVPP)	409(416)	455	487	448

All these data suggest that B3LYP (and to some extent also PBE0) is insufficient for the description of the conjugation effects in extended π systems. The insufficiency of standard functionals in such cases has been reported before,^16^ and it has been pointed out that inclusion of exact Hartree–Fock (HF) exchange is decisive in this context.^25^ Thus, it can be understood that the high nonlocality functional M06-2X provides the most stable results. In a similar sense, it was reported that the range-separated CAM-B3LYP functional outperformed the PBE0 functional in the case of delocalized excited states.^20^ To estimate basis set effects, the TDDFT calculations were carried out with the enhanced 6-311++G** basis set. These calculations led to a general modest redshift of about 25 nm (0.1 eV) for all systems and functionals considered. However, considering that this shift is rather small and uniform, it appears that the SVP basis set is sufficient, given that the resulting excitation energies are generally somewhat higher than those in the complete basis set limit. DMA-1T was also treated at the RI-CC2/QZVPP level, and the results showed a slight decrease in excitation energy relative to the TZVPP results. In summary, at this stage it can be concluded that the M06-2X functional is in excellent agreement with the RI-CC2 reference. To test the applicability of the developed strategy on “real-life materials” and to obtain a rough approximation of the computational time, we included one derivative of substance class BRA-*n*T (Figure 1), namely, BHA-1T (**4**; R=H, *n*=1), in our performance study to serve as a representative example. In this case only, the M06-2X functional was considered and good agreement with the RI-CC2/TZVPP reference was obtained. Comparison to DMA-1T shows that the outer phenyl groups have a small effect on the fluorescence, and they result in a redshift of about 25 nm (0.17 eV).

**Figure 5:**
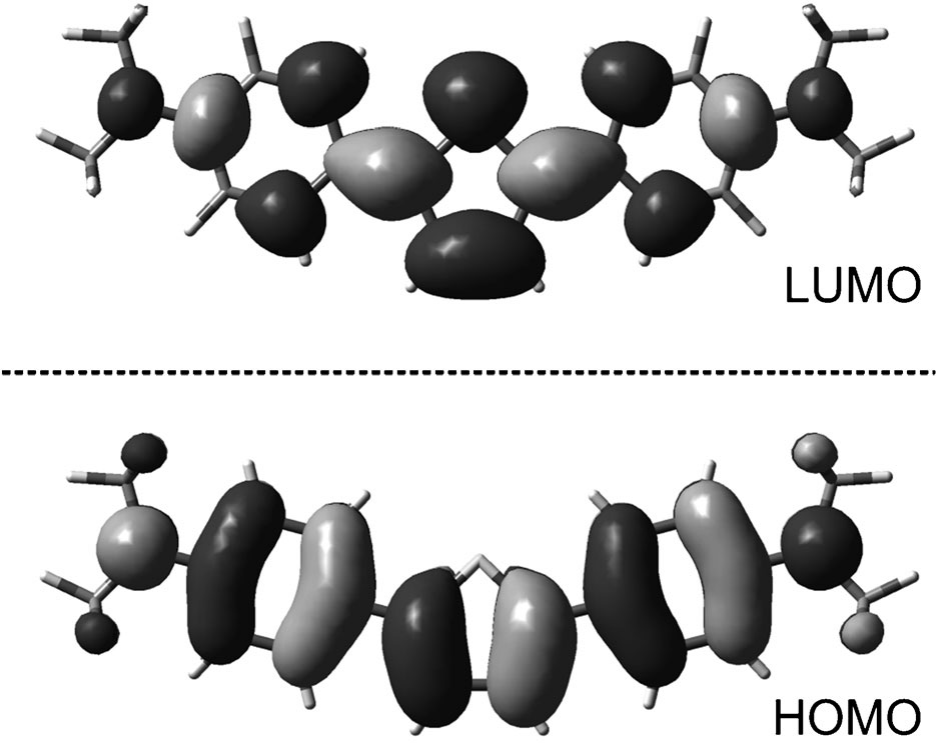
Molecular orbital plots (DMA-1T, S_1_, M06-2X/LR-PCM, THF).

**Figure 6:**
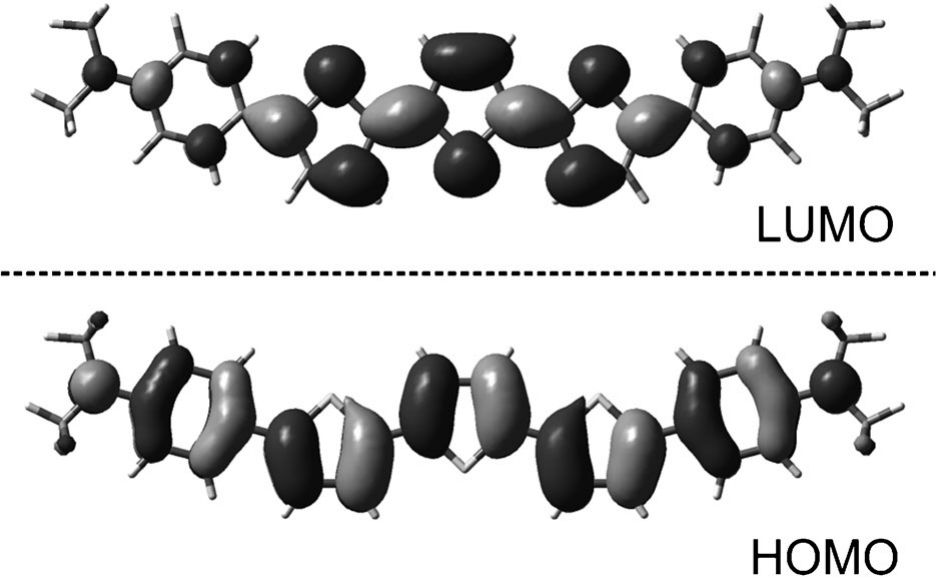
Molecular orbital plots (DMA-3T, S_1_, M06-2X/LR-PCM, THF).

Selected molecular orbital plots at the M06-2X level are shown in Figures 5 and 6; a larger set of orbitals, also including information on the other functionals, is given in Section C of the Supporting Information. In all cases, the main contribution to the excited state is a transition between the highest occupied molecular orbital (HOMO) and the lowest unoccupied molecular orbital (LUMO). These are always distributed over the whole planar system, which also includes the phenyl rings and the terminal amino nitrogen atoms. In the cases of larger systems, contributions of the outer phenyl rings to the LUMO are somewhat diminished. The HOMO of BHA-1T also contains some contributions from the outer phenyl rings. When comparing the shapes of the orbitals between the three functionals for the DMA-*n*T series, it is observed that these are virtually indistinguishable. However, an interesting difference between the functionals is observed when considering the weight of the HOMO/LUMO transition. In calculations with the B3LYP and PBE0 functionals, the HOMO/LUMO transition contributes with more than 99 % for the whole DMA-*n*T series (for B3LYP, this value was in most cases even above 99.9 %). By contrast, in the case of M06-2X this value steadily decreased along the series giving 98, 97, and 95 % for *n*=1, 2, and 3, respectively, with the HOMO−1/LUMO+1 transition coming into play. By comparison, the RI-CC2/TZVPP values are 95, 95, and 94 %, respectively. Some of us have recently discussed such properties,^21^ which are related to the concept of natural transition orbitals.^22^ In particular, it should be noted that additional transitions coming into play are related to electron correlation^23^ and entropy.^24^ An interpretation in the present context is that a pure HOMO/LUMO transition represents a homogeneous excited state, or in other words, an unbound exciton. Additional structures arising from electron–hole interactions can only be included through additional independent transitions. In this sense, it is expected that for bound excitons, the weight of the HOMO/LUMO transition should decrease as the size of the system is increased. Specifically, it has been pointed out that the amount of HF exchange is important for providing bound excitons.^25^ In agreement with this interpretation, the best results are obtained with M06-2X containing, at 54 %, a significantly larger proportion of HF exchange relative to that of the other functionals. In summary, we interpret our results in the sense that for the system sizes considered here (i.e. up to five conjugated rings), new excitonic effects start to come into play. In spite of their good performance for smaller molecules, the B3LYP and PBE0 functionals are incapable of describing these correctly. By contrast, M06-2X appears to provide a very good description for molecules of the size considered here. Furthermore, for even larger conjugated systems, inclusion of full HF exchange (at least after range separation) may be necessary.

As the next step, the fluorescence spectra in solution were considered. These data are, in particular, interesting, as it has been shown (e.g. for BMA-*n*T)^4^ that the photoluminescence spectra of solutions are similar to those recorded for the fabricated OLED. To investigate the influence of the solvent, three different solvents, namely, *n*-heptane (HEP), dichloromethane (DCM), and tetrahydrofuran (THF) were selected. The fluorescence spectra in these solvents were measured. Computationally, solvent effects were included at the PCM level in its linear response (LR) and state specific (SS) formulations (see Computational Details). The main effect, a redshift resulting from an increase in the solvent polarity, could be reproduced with all computational models.

**Figure 7:**
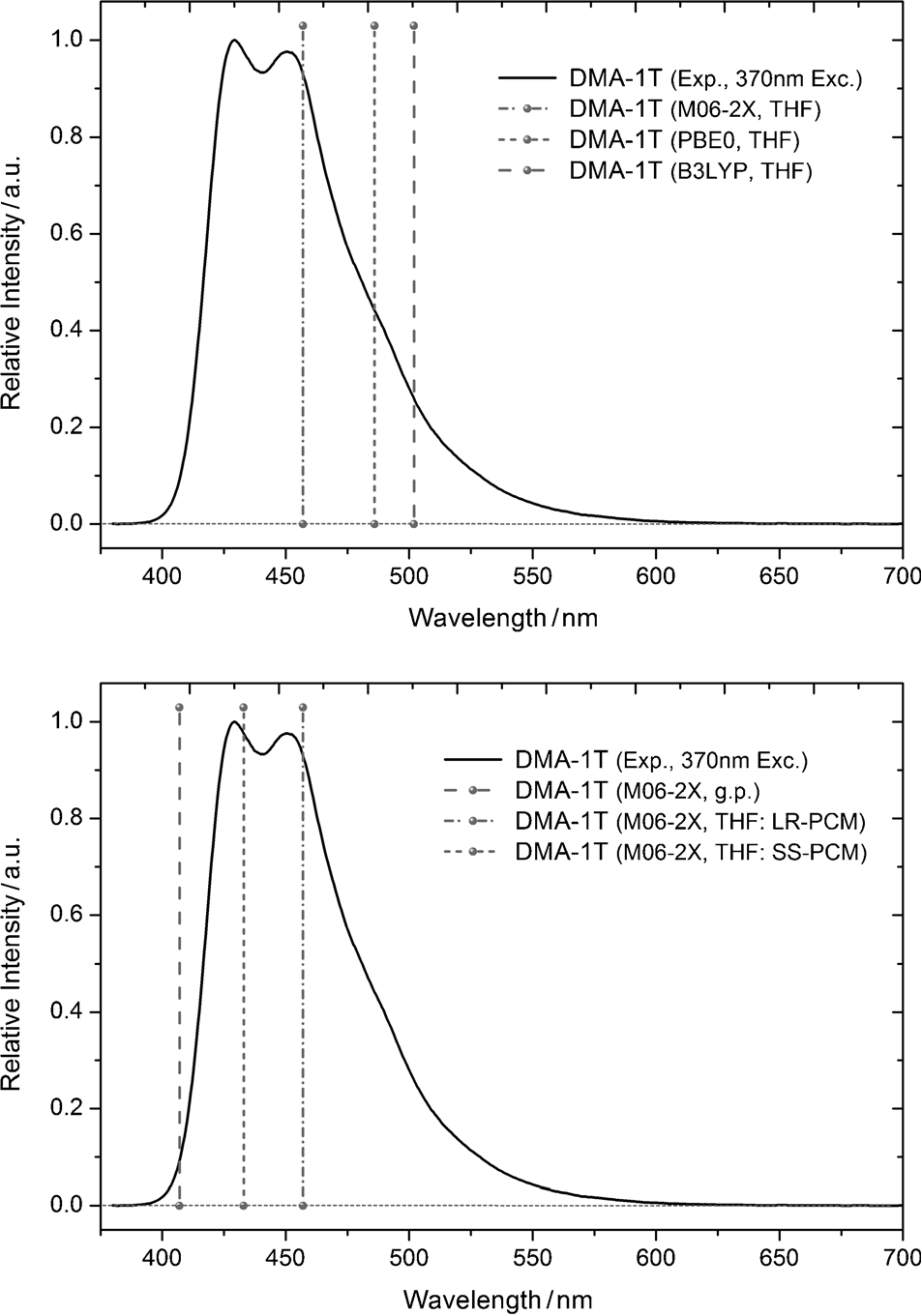
Vertical emissions of DMA-1T in THF calculated by using the B3LYP, PBE0, and M06-2X functionals and LR-PCM as solvent correction (top). Vertical emissions of DMA-1T calculated by using the M06-2X functional in the gas phase and in THF solution by applying LR-PCM or SS-PCM (bottom). Experimental spectra measured from THF solutions.

Further results can be extracted from Table 3: 1) The above-mentioned trend in emission wavelengths (B3LYP> PBE0> M06-2X) remains true if solvent corrections are applied; to illustrate this, the LR-PCM vertical emissions calculated by using the three different functionals are superimposed on the experimental spectrum in Figure 7 (top). 2) M06-2X, in particular with SS-PCM, provides very reliable results; values obtained from both B3LYP and PBE0 are redshifted by several tens of nanometers for both LR-PCM and SS-PCM corrections. 3) For all functionals, LR-PCM overestimates the solvatochromic redshift, whereas the SS-PCM results are very close to the experimental values. A more-detailed discussion of this overestimation and additional information on these solvation models are given in ref. 19c. Furthermore, it is worth mentioning that oscillator strengths (*f*) increase quite strongly when moving from DMA-1T to DMA-3T (e.g. M06-2X/SS-PCM in THF: 1.40, 1.77, and 2.13 for DMA-1T, DMA-2T, and DMA-3T, respectively). In all cases, the transition dipole vector lies in the molecular plane and points along the aromatic backbone.

**Table 3:** Comparison of calculated vertical emissions by applying SS-PCM (LR-PCM results in parentheses) in different solvents and experimental (exp.) values. All data denoted in nm

Parameter		Vertical emission^[a]^			
Solvent	Functional	DMA-1T	DMA-2T	DMA-3T	BHA-1T
HEP	B3LYP	452(467)	524(544)	582(604)	–
	PBE0	438(453)	506(524)	558(579)	–
	M06-2X	417(429)	470(486)	506(523)	–
	exp.	413–437	n.a.^[b]^	n.a.^[b]^	–
					
DCM	B3LYP	471(505)	555(596)	624(667)	–
	PBE0	458(489)	536(572)	599(635)	–
	M06-2X	434(461)	495(526)	534(567)	–
	exp.	438–458	490–511	525–548	–
					
THF	B3LYP	470(502)	552(592)	621(663)	–
	PBE0	457(486)	534(569)	595(631)	–
	M06-2X	433(457)	493(523)	532(564)	455(474)
	exp.	429–451	481–507	517–543	444–468

[a] All experimental spectra showed two distinct maxima. [b] Substance insoluble in *n*-heptane.

**Figure 8:**
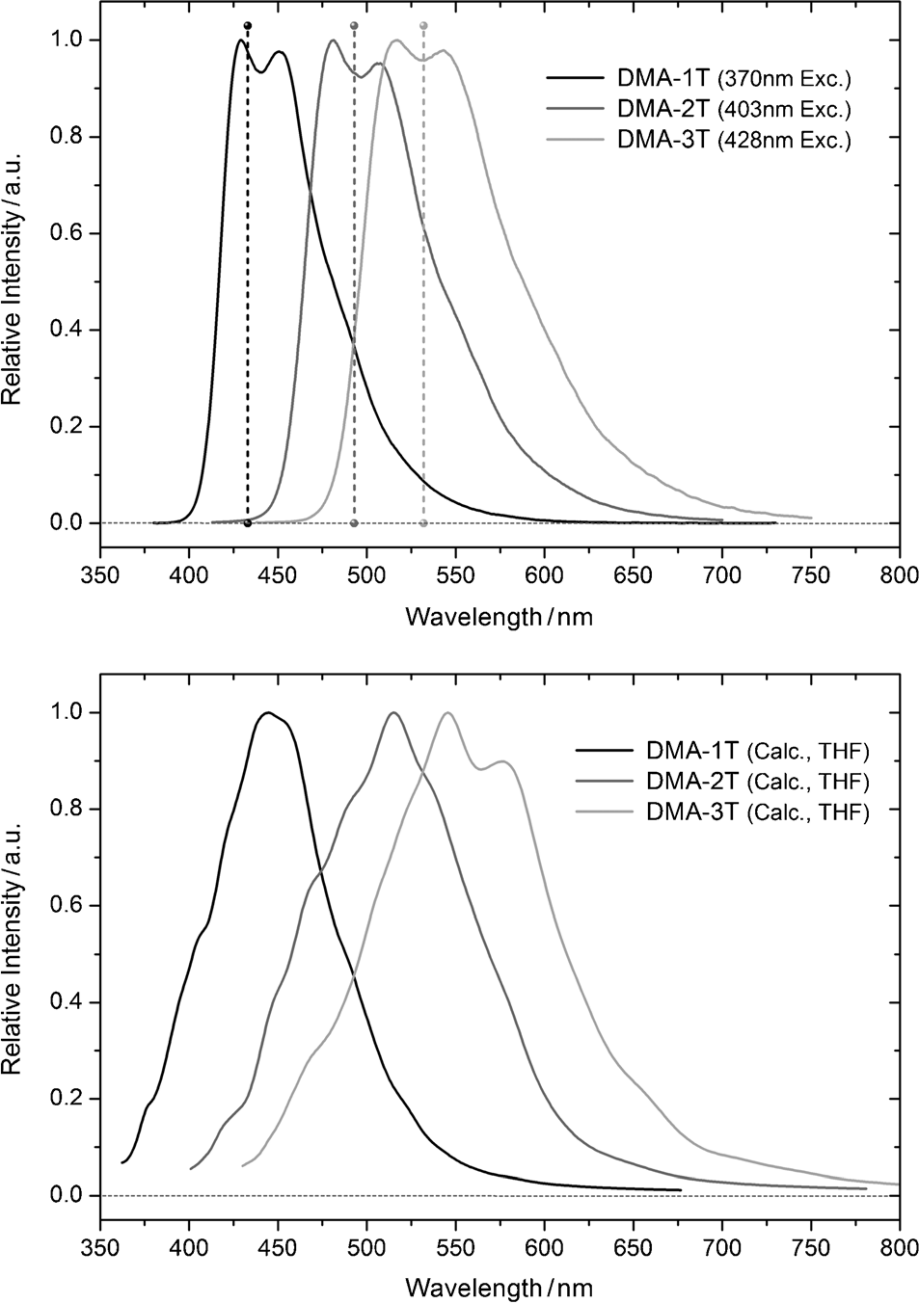
Experimental (top) and simulated (M06-2X, LR-PCM, bottom) emission spectra of DMA-*n*T (*n*=1-3) in THF. Vertical emissions (M06-2X, SS-PCM) are indicated by dashed lines.

For a graphical illustration of the obtained accuracy, Figure 7 (bottom) shows the experimental fluorescence spectrum of DMA-1T (THF solution) as well as the M06-2X/SVP-based theoretical values obtained when applying no (gas phase) LR-PCM or SS-PCM for the influence of the solvent. The gas-phase value is strongly blueshifted relative to both maxima of the experimental spectrum. It lies close to the onset of the spectrum, that is, the experimental value for the 0–0 transition, which should lie at significantly higher energies than the vertical fluorescence. By contrast, the LR-PCM value is redshifted to such an extent that it is situated at higher wavelengths with respect to both experimental emission maxima. The most accurate prediction for the vertical emission is obtained when the SS-PCM correction is applied. Considering Table 3, this method yields a value that is situated between the two maxima of the experimental spectrum in all cases except DMA-1T in DCM, in which it is just below them. Figures 8 (top) and 9 show the experimental spectra and the calculated vertical emissions for DMA-*n*T and BHA-1T, respectively, highlighting the suitability of the applied computational models.

The next step in the course of this study was to examine the band broadening of the fluorescence spectrum. In addition to recording the experimental spectra, simulations were performed by a semiclassical approach as outlined in the Computational Details Section. For reasons of computational efficiency and ease of implementation, spectra simulations were performed by applying a LR-PCM. As outlined above, this leads to a slight redshift in the simulated spectra. However, the band shape should be affected to a lesser extent. The experimental spectra for the DMA-*n*T series are illustrated in Figure 8 (top). All three spectra are similar in appearance with a slightly noticeable vibronic structure featuring peaks that are, in all cases, separated by about 1400 cm^−1^. This feature probably arises from aromatic stretching modes, considering that the corresponding internal coordinates are strongly altered during the excited-state structural relaxation (cf. Table 1). Such modes around 1400 cm^−1^ were indeed found in the ground-state normal mode analysis of these molecules. In contrast to the fluorescence spectra, the S_1_ peaks in the absorption spectra of these molecules did not show any vibronic structures (see Section D of the Supporting Information). This difference most probably originates from the planarization in the excited state, as described above, and a concomitant increase in the torsional barrier (cf. ref. 19b). The full widths at half maximum (FWHM) for the experimental spectra are 0.40, 0.38, and 0.39 eV for DMA-1T, DMA-2T, and DMA-3T, respectively. The simulated spectra (Figure 8, bottom) are in acceptable agreement with the experimental ones with respect to the position of the maximum as well as the broadening of the band. However, the FWHM broadening of these spectra (0.55, 0.53, and 0.48 eV) is somewhat too large relative to the experimental ones. A notable difference can be seen at the blue edge, at which point the experimental spectra decrease more sharply than their computational counterparts. These differences may be related to the treatment of torsion, which is, by default, simply considered as a harmonic oscillator in Cartesian coordinates. Considering that, in our semiclassical approach, only the modes of the planar excited-state structure are included explicitly, this still gives reasonable results with the agreement shown above. By contrast, in a Franck–Condon approach, in which the wavefunctions of low-frequency torsional modes over multiple nonplanar wells in the ground state also have to be computed, the situation becomes more difficult and requires special techniques or artificial planarization of the molecule.19c, 26 A rigorous approach in which all of the torsions are considered as hindered rotors in curvilinear coordinates is, in principle, also possible in our approach. However, such a treatment was already quite involved for the smaller bithiophene system, which contains only one torsion,19c, 26 and therefore this treatment is beyond the scope of this work.

It should be noted that the semiclassical approach used here is only capable of reproducing the spectral envelope without vibronic progressions. The structure that is visible in these spectra is probably only of stochastic nature and would disappear with increased sampling (requiring significantly enhanced computational effort). However, the presented computational protocol carries the potential to provide interesting additional information in the event that either decomposition of the spectra is performed (cf. ref. 27) or the involved modes are analyzed in more detail. In particular, it would be of great interest to understand which modes comprise the principle components of spectral broadening, considering that such information could be used to modify the spectral properties in a rational design process. Such an analysis, which is planned as a future project, should also shed new light on the differences with regard to the experimental results at the spectral edges.

**Figure 9:**
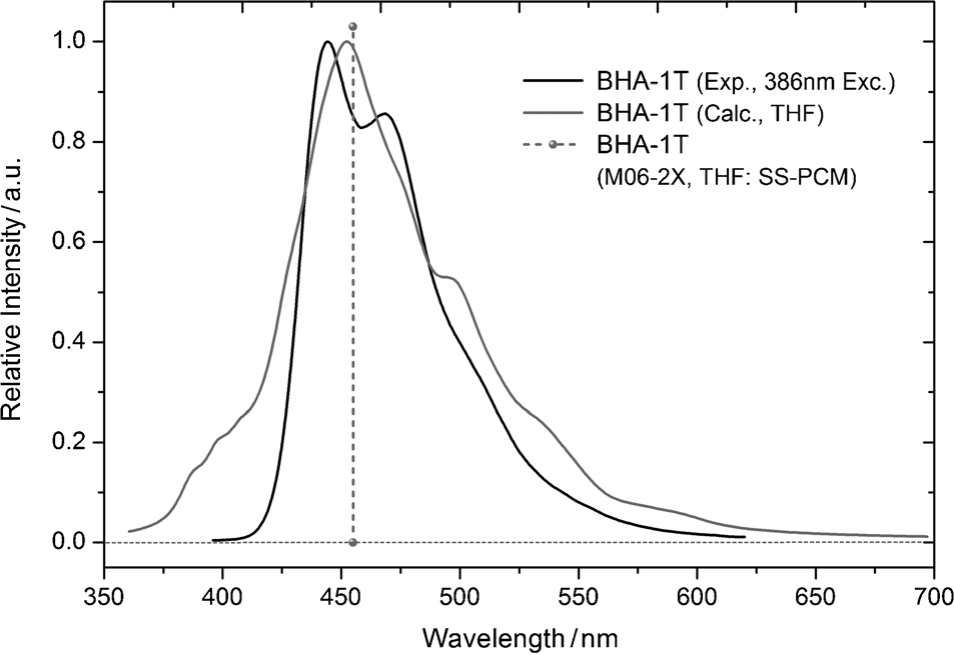
Experimental and simulated (M06-2X, LR-PCM) emission spectra of BHA-1T in THF. Vertical emission (M06-2X, SS-PCM) is indicated by a dashed line.

Figure 9 summarizes the whole protocol as applied to BHA-1T. In this case, good agreement is again found. The vertical emission (M06-2X, SS-PCM) is located between the two maxima of the experimental spectrum, which is reproduced by the spectra simulation over a wide range, except for the blue onset (M06-2X, LR-PCM).

## 3. Conclusions

The aim of this study was to establish a computational protocol for the prediction of the electronic emission spectra of functional organic molecules. Oligothiophene-based compounds, widely applied in the field of organic electronics, were characterized with regard to their fluorescent properties. Three representative model compounds were synthesized, analyzed, and used to benchmark some critical parameters for the calculations. Beyond various functionals under investigation, M06-2X gave highly accurate results in comparison to both experiment and RI-CC2 benchmark calculations, whereas for B3LYP and to a lesser extent PBE0, the values were redshifted, especially when applied to molecules with extended conjugated π systems. A tentative explanation of this phenomenon was given in terms of excitonic structure starting to arise in systems of this size. Solvent effects were included by applying a PCM in its linear response and state specific formulations, and the latter provided superior results. An accuracy of approximately ±15 nm can be expected for the prediction of experimental fluorescence maxima in solution by calculating vertical emissions. The same satisfactory results can be obtained also for larger molecules of immediate interest, for example, BHA-1T. Moreover, vibrational broadening was simulated in solution by utilizing the framework of the Newton-X software. The obtained spectra are generally in good agreement with experimental data. However, an overestimation of the blue onset could be observed, and this in turn leads to a somewhat increased spectral width. Investigation into possible reasons leading to this effect will be a matter for future studies.

Having verified the utility of this computational protocol, the next task will be to obtain more-detailed insight into the underlying physics, and its influence on the spectral properties of these compounds, which will be achieved by extended analysis of our data. Aside from considering influences on the position of the absorption maximum, a particular focus will also be on the molecular modes affecting spectral width. On the basis of this understanding, new molecules with desired properties can be designed and synthesized and their properties determined. We believe that the close connection between synthesis, spectroscopy, and computation, which is provided by our laboratory, provides an ideal environment for this feedback cycle.

## Computational and Experimental Details

### General Methods

Density functional theory (DFT) and time-dependent (TD) DFT calculations were performed by using three different functionals: B3LYP,^17^ PBE0,6a, 18 and M06-2X.6b Most TDDFT calculations were performed with the polarized double-ζ SVP basis set.^28^ In the cases specified, the larger 6-311++G** basis set^29^ was used as a reference. Geometry optimizations were performed without symmetry constraints and all ground- and excited-state minima were verified by normal mode analysis. Solvent effects were included through the polarizable continuum model (PCM)^30^ in its linear response (LR-PCM)^31^ and state specific (SS-PCM)^7^ formulations, always considering the equilibrium time regime (eq) for the excited state. Vertical fluorescence energies were obtained after geometry optimizations in the S_1_ state at the TDDFT level, both in the gas phase and by using LR-PCM (eq). For benchmark purposes, higher-level calculations with the use of the second-order approximate coupled cluster method (CC2)^32^ with resolution of the identity approximation^33^ (RI-CC2) were performed as well by using the TZVPP and QZVPP basis sets.^34^ Subsequently, vibrational broadening of the spectra was simulated by using a Wigner distribution formalism, as described in ref. 35, by applying a phenomenological broadening of *δ*=0.1 eV. Taking the output of the frequency computations as the source of the normal modes, the Newton-X framework is capable of fulfilling this task very effectively. Knowledge upon the excited-state normal modes is used to generate a set of nonequilibrium geometries with a selectable number of data points, which in these particular cases was 500 (DMA-*n*T) or 250 (BHA-1T). At each point, the S_1_→S_0_ excitation energy was calculated by using the M06-2X functional at the TDDFT level of theory. Considering that these are all independent single-point energy calculations, this step can be easily performed in parallel by distributing them to a large number of compute nodes. After computation of all emission energies, the data is collected to construct a vibrationally broadened spectrum. In the case of solution-phase spectra, not only the emission energies but also the geometries and frequencies were computed in solution by applying LR-PCM (eq). In addition to the semiclassical spectra, a Franck–Condon (FC) simulation of the emission spectrum of DMA-1T was attempted in Gaussian 09. This led to the problem of an almost vanishing overlap integral between the two vibrational ground states of the S_0_ and S_1_ states, and thus the calculation was aborted. A solution to this problem requires either an artificial planarization of the relevant torsional coordinates or a more-involved treatment of these coordinates (cf.19c, 26) and was not attempted. All TDDFT computations were performed using the Gaussian 09 package, revision A.02.^36^ RI-CC2 calculations were carried out with Turbomole 6.3.^37^ Spectra simulations were performed in the framework of Newton-X.^35^, ^38^ An interface of Newton-X to Gaussian 09, which was developed in the course of the project, is available in the current Newton-X release.

If not stated otherwise, chemicals were obtained from commercial sources and used without further purification. Column chromatography was performed on silica 60 (Merck, 40-63 μm) by using distilled solvents as given. The following substances were synthesized according to the literature: 5,5′-dibromo-2,2′-bithiophene (**1 b**),^39^ 5,5′′-dibromo-2,2′:5′,2′′-terthiophene (**1 c**),^40^ *N*,*N*-dimethyl-4-(4,4,5,5-tetramethyl-1,3,2-dioxaborolan-2-yl)benzeneamine (**2 a**),^41^ and *N*,*N*-diphenyl-4-(4,4,5,5-tetramethyl-1,3,2-dioxaborolan-2 L)benzeneamine (**2 b**).41b, 42

NMR spectra were recorded at 400 MHz for ^1^H and at 100 MHz for ^13^C with a Bruker Avance DRX-400 spectrometer. Data for ^1^H NMR are reported as follows: chemical shift in parts per million from tetramethylsilane with the residual solvent signal as an internal reference (CD_2_Cl_2_: *δ*=5.32 ppm, [D_1_]TFA: *δ*=11.50 ppm; TFA=trifluoroacetic acid), multiplicity (s=singlet, d=doublet, t=triplet, and m=multiplet), coupling constant, and integration. ^13^C NMR spectra are reported in ppm from tetramethylsilane by using the central peak of the solvent as reference (CD_2_Cl_2_: *δ*=54.0 ppm, [D_1_]TFA: *δ*=116.6 ppm), multiplicity with respect to proton (deduced from APT experiments, s=quaternary C, d=CH, t=CH_2_, q=CH_3_). Laser desorption ionization (LDI) high-resolution (HR) mass spectra were obtained with a Synapt HDMS (Waters, U.K.). Experimental fluorescence characterizations were performed with an Edinburgh FLS920 fluorometer; signal processing was realized by using Savitzky–Golay methods. Single-crystal X-ray diffraction data were recorded with a Bruker Kappa APEXII CCD diffractometer, *λ*(MoKα)=0.71073 Å. Refinement of *F*^2^ was done by using the *SHELXTL* program (version 2008, Bruker AXS, Inc., Madison, WI).

### Syntheses

General procedure for the synthesis of DMA-*n*T and BHA-1T compounds: KO*t*Bu was added (3.0 mmol) to a suspension of dibromothiophene **1 a**–**c** (1.0 mmol) and boronic ester **2 a**,**b** (3.0 mmol) in degassed *i*PrOH/H_2_O (v/v=3/1, 12 mL). Subsequently, under an argon atmosphere a solution of (IPr)Pd(allyl)Cl13a (2.0 mol % for **3 a**–**c**, 5.0 mol % for **4**) was injected into the stirred mixture by syringe and heated to reflux (≍80 °C). Upon completion of the reaction (monitored by TLC, 1–2 h), the products were purified by chromatography or crystallization. Analytical samples of **3 a**–**c** and **4** were (re)crystallized from pyridine or cyclohexane, respectively, prior photophysical characterization.

4,4′-(2,5-Thiophenediyl)bis[*N*,*N*-dimethylbenzeneamine] (**3 a**, DMA-1T): The reaction mixture was poured into H_2_O/CHCl_3_ (1:1), and the aqueous layer was extracted with CHCl_3_. The combined organic layers were dried with Na_2_SO_4_ and concentrated under reduced pressure. Flash chromatography (cyclohexane/DCM, 6→100 %) on triethylamine-preconditioned silica gel yielded **3 a** (241 mg, 75 %) as an orange-brown solid. ^1^H NMR (400 MHz, [D_1_]TFA): *δ*=7.98 (d, *J*=8.4 Hz, 4 H), 7.68 (d, *J*=8.4 Hz, 4 H), 7.52 (s, 2 H), 3.53 ppm (s, 12 H); ^13^C (100 MHz, [D_1_]TFA): *δ*=150.5 (s), 142.8 (s), 126.7 (d), 123.4 (s), 122.0 (d), 113.0 (d), 40.8 ppm (q); HRMS (LDI-qTOF): calcd for C_20_H_22_N_2_S [M]^+^ 322.1504; found 322.1632.

4,4′-[2,2′-Bithiophene-5,5′-diyl]bis[*N*,*N*-dimethylbenzeneamine] (**3 b**, DMA-2T): The reaction was performed following the general procedure. Upon completion, the reaction mixture was filtered through a sintered funnel, and the residue was washed with *i*PrOH/H_2_O (3:1), EtOH, and light petroleum ether and then dried under vacuum. Crystallization from pyridine yielded **3 b** as a light orange solid (295 mg, 73 %). ^1^H NMR (400 MHz, CD_2_Cl_2_+[D_1_]TFA): *δ*=7.79 (d, *J*=8.6 Hz, 4 H), 7.58 (d, *J*=8.6 Hz, 4 H), 7.37 (d, *J*=3.9 Hz, 2 H), 7.28 (d, *J*=3.9 Hz, 2 H), 3.33 ppm (s, 12 H); ^13^C (100 MHz, CD_2_Cl_2_+[D_1_]TFA): *δ*=141.3 (s), 141.1 (s), 138.4 (s), 137.1 (s), 128.0 (d), 126.4 (d), 126.0 (d), 121.5 (d), 47.8 ppm (q); HRMS (LDI-qTOF): calcd for C_24_H_24_N_2_S_2_ [M]^+^ 404.1381; found 404.1393.

4,4′-[2,2′:5′,2′′-Terthiophene-5,5′′-diyl]bis[*N*,*N*-dimethylbenzeneamine] (**3 c**, DMA-3T): The reaction was carried out on a 0.5 mmol scale; the workup was performed in a manner identical to that for **3 b**. Crystallization from pyridine afforded **3 c** as an orange solid (160 mg, 66 %). ^1^H NMR (400 MHz, CD_2_Cl_2_+[D_1_]TFA): *δ*=7.78 (d, *J*=8.6 Hz, 4 H), 7.57 (d, *J*=8.6 Hz, 4 H), 7.36 (d, *J*=3.9 Hz, 2 H), 7.24 (d, *J*=3.9 Hz, 2 H), 7.21 (s, 2 H), 3.33 ppm (s, 12 H); ^13^C (100 MHz, CD_2_Cl_2_+[D_1_]TFA): *δ*=141.2 (s), 140.8 (s), 138.6 (s), 137.1 (s), 136.7 (s), 128.0 (d), 126.4 (d), 125.6 (d, 2 C), 121.5 (d), 47.8 ppm (q); HRMS (LDI-qTOF): calcd for C_28_H_26_N_2_S_3_ [M]^+^ 486.1258; found 486.1165.

4,4′-[2,5-Thiophenediyl]bis[*N*,*N*-diphenylbenzeneamine] (**4**, BHA-1T): Workup was performed in analogy to that performed for **3 a**. Flash chromatography (cyclohexane/DCM, 5→10 %) with silica gel yielded **4** (526 mg, 92 %) as a bright yellow solid; single crystals were obtained from cyclohexane. ^1^H NMR (400 MHz, CD_2_Cl_2_): *δ*=7.50 (d, *J*=8.4 Hz, 4 H), 7.32–7.24 (m, 8 H), 7.20 (s, 2 H), 7.15–7.01 ppm (m, 16 H); ^13^C (100 MHz, CD_2_Cl_2_): *δ*=148.0 (s), 147.8 (s), 143.1 (s), 129.9 (d), 128.9 (s), 126.7 (d), 125.1 (d), 124.2 (d), 123.7 (d), 123.7 ppm (d); HRMS (LDI-qTOF): calcd for C_40_H_30_N_2_S [M]^+^ 570.2130; found 570.1812. Crystal data (yellow prism of 0.40×0.30×0.20 mm from cyclohexane): C_40_H_30_N_2_S, FW=570.72, *T*=296(2) K, triclinic, space group *P*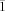, *a*=9.8178(2) Å, *b*=12.3368(2) Å, *c*=12.9148(2) Å, *α*=88.365(1)°, *β*=76.765(1)°, *γ*=78.962(1)°, *V*=1494.34(5) Å^3^, *Z*=2, ρ_calcd_=1.268 g cm^−3^, *μ*=0.141 mm^−1^. Of 31978 reflections measured up to *θ*=30°, 8659 were unique. Refinement of *F*^2^ concluded with *R*1=0.0659 and *wR*2=0.1450 for 388 parameters and all data. CCDC-858839 http://www.ccdc.cam.ac.uk/cgi-bin/catreq.cgicontains the supplementary crystallographic data for this paper. These data can be obtained free of charge from The Cambridge Crystallographic Data Centre via http://www.ccdc.cam.ac.uk/data_request/cif.

## Supplementary material

As a service to our authors and readers, this journal provides supporting information supplied by the authors. Such materials are peer reviewed and may be re-organized for online delivery, but are not copy-edited or typeset. Technical support issues arising from supporting information (other than missing files) should be addressed to the authors.
