# Supplementary material for: Synthesis, Spectroscopy, and Computational Analysis of Photoluminescent Bis(aminophenyl)-Substituted Thiophene Derivatives
Source: Chemphyschem. 2013 Feb 26;14(5):1016–24. doi: 10.1002/cphc.201201006 (PMC3644869; doi:10.1002/cphc.201201006)
Supplement: Supplementary file 1 [file cphc0014-1016-SD1.pdf]

CHEMPHYSCHEM

Supporting Information

© Copyright Wiley-VCH Verlag GmbH & Co. KGaA, 69451 Weinheim, 2013

Synthesis, Spectroscopy, and Computational Analysis of Photoluminescent Bis(aminophenyl)-Substituted Thiophene Derivatives

Daniel Lumpi,[a] Ernst Horkel,*[a] Felix Plasser,[b] Hans Lischka,[c] and Johannes Fröhlich[a]

cphc_201201006_sm_miscellaneous_information.pdf

A. OPTIMIZED GEOMETRIES

A.1. OPTIMIZED GEOMETRIES OF DMA-2T, IMAGES

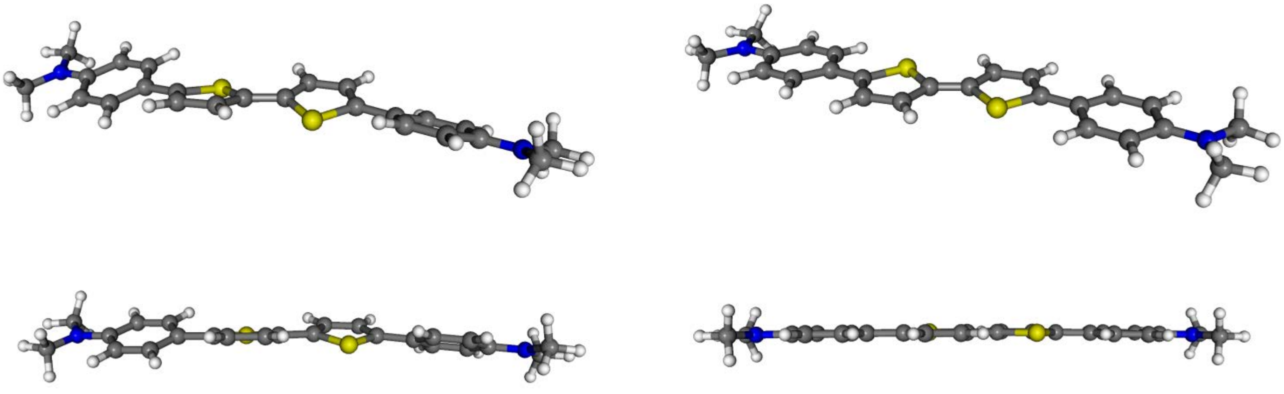

(a) (b)

Figure S1. Optimized geometries of DMA-2T (M06-2X/SVP, gas phase). S_0 (a); S_1 (b)

A.2. OPTIMIZED GEOMETRIES OF DMA-3T, IMAGES

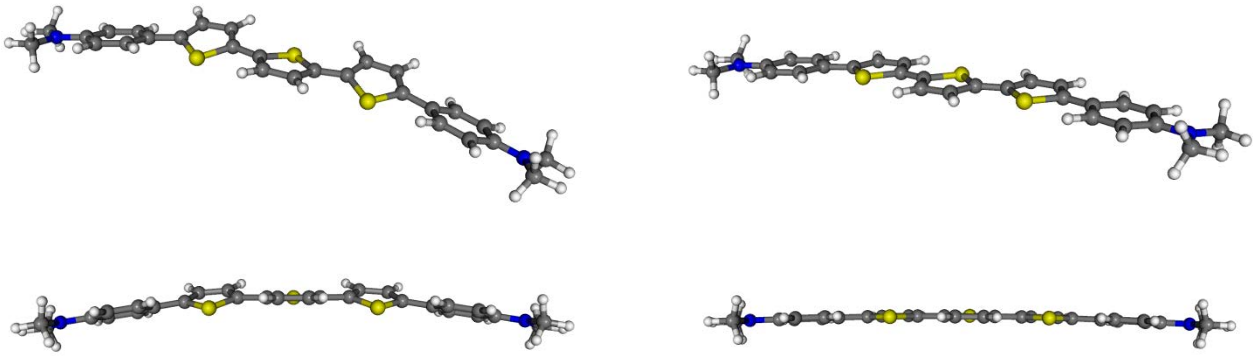

(a) (b)

Figure S2. Optimized geometries of DMA-3T (M06-2X/SVP, gas phase). S_0 (a); S_1 (b)

A.3. OPTIMIZED GEOMETRIES OF BHA-1T, IMAGES

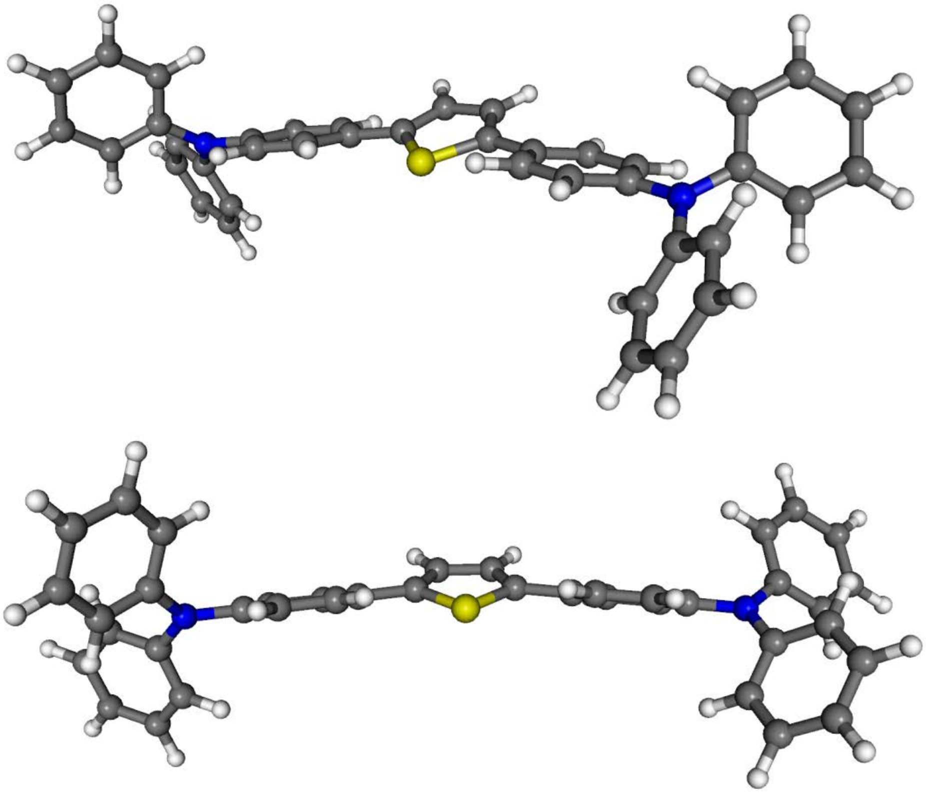

(a)

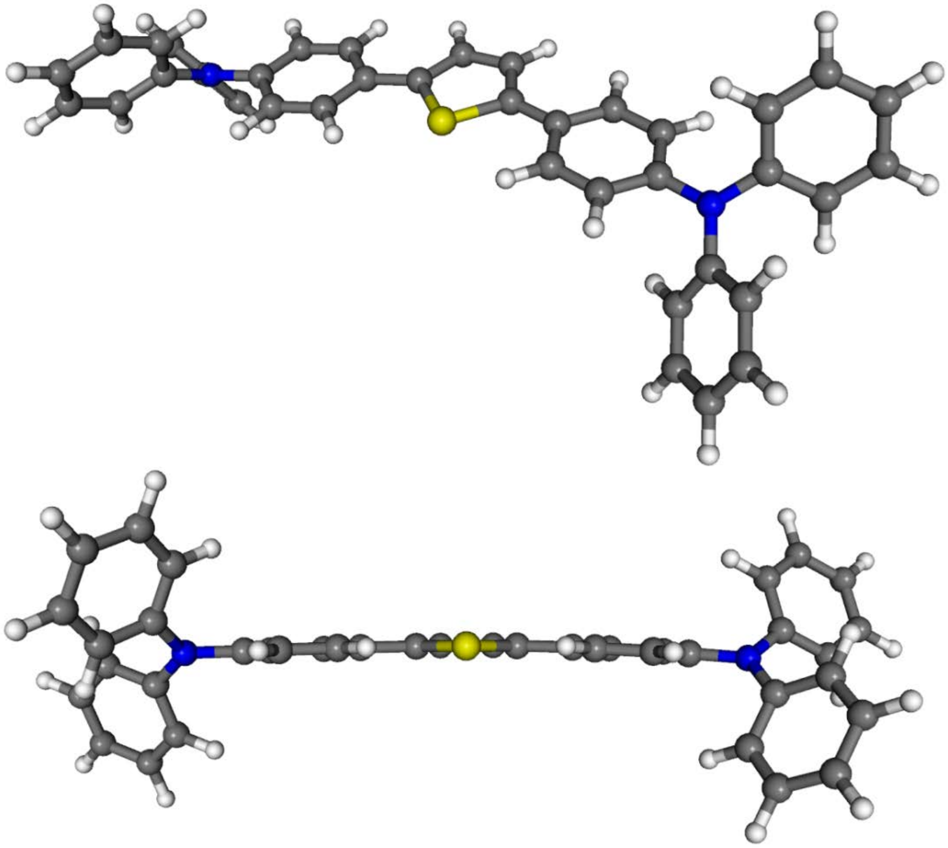

(b)

Figure S3. Optimized geometries of BHA-1T (M06-2X/SVP, gas phase). S_0 (a); S_1 (b)

B. OPTIMIZED GEOMETRIES, ATOMIC COORDINATES

B.1. OPTIMIZED GEOMETRY S_0 OF DMA-1T

```
45
XYZ file generated by gabedit : coordinates in Angstrom
C          1.2575760000         -0.8072230000         -0.1680200000
C          0.7111290000         -2.0304330000         -0.4761470000
C         -0.7111320000         -2.0304320000         -0.4761440000
C         -1.2575760000         -0.8072220000         -0.1680130000
S          0.0000010000          0.3473320000          0.1448800000
C         -2.6720860000         -0.4246750000         -0.0822190000
C          2.6720870000         -0.4246780000         -0.0822330000
C          3.0904730000          0.9045930000         -0.2337460000
C          4.4281680000          1.2665350000         -0.1616260000
C          5.4320370000          0.2981630000          0.0608560000
C          5.0121560000         -1.0421430000          0.2159410000
C          3.6689590000         -1.3835900000          0.1498010000
C         -3.0904750000          0.9045900000         -0.2337770000
C         -4.4281700000          1.2665330000         -0.1616490000
C         -5.4320340000          0.2981690000          0.0608920000
C         -5.0121490000         -1.0421310000          0.2160140000
C         -3.6689520000         -1.3835790000          0.1498660000
N          6.7626300000          0.6454080000          0.1262910000
C          7.7589760000         -0.3741160000          0.3423520000
C          7.1492430000          2.0257970000         -0.0284490000
N         -6.7626260000          0.6454140000          0.1263470000
C         -7.1492580000          2.0257800000         -0.0285570000
C         -7.7589710000         -0.3741190000          0.3423660000
H          1.3165450000         -2.8988040000         -0.7347410000
H         -1.3165520000         -2.8988020000         -0.7347360000
H          2.3475510000          1.6818680000         -0.4276230000
H          4.6901290000          2.3149000000         -0.2908610000
H          5.7408350000         -1.8289430000          0.4023790000
H          3.3847770000         -2.4264500000          0.3022180000
H         -2.3475570000          1.6818570000         -0.4276990000
H         -4.6901340000          2.3148920000         -0.2909260000
H         -5.7408250000         -1.8289270000          0.4024860000
H         -3.3847670000         -2.4264340000          0.3023150000
H          7.6207720000         -0.8898160000          1.3088160000
H          7.7425180000         -1.1405620000         -0.4518670000
H          8.7530960000          0.0868550000          0.3456440000
H          6.6983050000          2.6695420000          0.7466730000
H          8.2385620000          2.1081210000          0.0563240000
H          6.8531410000          2.4293270000         -1.0125830000
H         -8.2385730000          2.1081050000          0.0562500000
H         -6.8531960000          2.4291940000         -1.0127520000
H         -6.6982990000          2.6696220000          0.7464700000
H         -7.6207560000         -0.8898560000          1.3088070000
H         -8.7530900000          0.0868540000          0.3456960000
H         -7.7425250000         -1.1405350000         -0.4518840000
```

Table S1. Optimized geometry S_0 of DMA-1T (M06-2X/SVP, gas phase).

B.2. OPTIMIZED GEOMETRY S_1 OF DMA-1T

```
45
XYZ file generated by gabedit : coordinates in Angstrom
C        -1.2676350000         0.8022750000        -0.0249960000
C        -0.6894200000         2.1029480000        -0.0153070000
C         0.6894300000         2.1029430000        -0.0153110000
C         1.2676360000         0.8022660000        -0.0249970000
S        -0.0000040000        -0.4243080000        -0.0408300000
C         2.6366250000         0.4395540000        -0.0179780000
C        -2.6366290000         0.4395740000        -0.0179750000
C        -3.0579620000        -0.9235240000        -0.0273670000
C        -4.3899070000        -1.2788530000        -0.0146990000
C        -5.4151230000        -0.2978410000         0.0081130000
C        -5.0086980000         1.0651940000         0.0149840000
C        -3.6771870000         1.4157610000         0.0023390000
C         3.0579460000        -0.9235460000        -0.0273700000
C         4.3898890000        -1.2788880000        -0.0147010000
C         5.4151140000        -0.2978800000         0.0081110000
C         5.0086990000         1.0651540000         0.0149820000
C         3.6771910000         1.4157330000         0.0023360000
N        -6.7432980000        -0.6485920000         0.0236270000
C        -7.7587730000         0.3753150000         0.0478260000
C        -7.1171930000        -2.0417150000         0.0216810000
N         6.7433000000        -0.6485900000         0.0236270000
C         7.1172820000        -2.0416910000         0.0216860000
C         7.7587090000         0.3753800000         0.0478280000
H        -1.2924240000         3.0096110000        -0.0078090000
H         1.2924400000         3.0096010000        -0.0078170000
H        -2.3049030000        -1.7142460000        -0.0449830000
H        -4.6438950000        -2.3376600000        -0.0225010000
H        -5.7547720000         1.8582330000         0.0321160000
H        -3.4172430000         2.4750110000         0.0096670000
H         2.3048810000        -1.7142610000        -0.0449890000
H         4.6438590000        -2.3376960000        -0.0225030000
H         5.7547860000         1.8581840000         0.0321150000
H         3.4172580000         2.4749850000         0.0096640000
H        -7.6772000000         1.0144140000         0.9444210000
H        -7.7012880000         1.0316880000        -0.8379620000
H        -8.7476890000        -0.0961450000         0.0565790000
H        -6.7172530000        -2.5738290000         0.9025120000
H        -8.2093420000        -2.1238150000         0.0411100000
H        -6.7499090000        -2.5632360000        -0.8794610000
H         8.2094360000        -2.1237210000         0.0411150000
H         6.7500320000        -2.5632390000        -0.8794540000
H         6.7173780000        -2.5738280000         0.9025190000
H         7.6770900000         1.0144740000         0.9444240000
H         8.7476570000        -0.0960130000         0.0565800000
H         7.7011770000         1.0317500000        -0.8379590000
```

Table S2. Optimized geometry S_1 of DMA-1T (M06-2X/SVP, gas phase).

B.3. OPTIMIZED GEOMETRY S_0 OF DMA-2T

```
52
XYZ file generated by gabedit : coordinates in Angstrom
C        -3.1625950000         0.5625800000        -0.1081790000
C        -2.4808520000         1.7435540000        -0.2899140000
C        -1.0701230000         1.5815210000        -0.3224420000
C        -0.6725520000         0.2742100000        -0.1644420000
S        -2.0482800000        -0.7591760000         0.0501750000
C        -4.6110830000         0.3423810000        -0.0300100000
C        -5.4750220000         1.3757990000         0.3604680000
C        -6.8492300000         1.1979160000         0.4197020000
C        -7.4371980000        -0.0463920000         0.0972810000
C        -6.5670160000        -1.0907550000        -0.2872250000
C        -5.1951020000        -0.8915430000        -0.3488940000
N        -8.7988000000        -0.2332690000         0.1579410000
C        -9.3608430000        -1.5140270000        -0.1944170000
H       -10.4503860000        -1.4727420000        -0.0864650000
H        -9.1318850000        -1.7939820000        -1.2378080000
H        -8.9857490000        -2.3200350000         0.4593250000
C         9.3605080000         1.5147490000        -0.1939070000
H        10.4499050000         1.4741990000        -0.0842350000
H         9.1329340000         1.7944720000        -1.2376810000
H         8.9838850000         2.3205390000         0.4591940000
C        -9.6571410000         0.8692830000         0.5150880000
C         9.6573080000        -0.8688540000         0.5144100000
H       -10.7007070000         0.5358670000         0.4965500000
H        -9.4411190000         1.2479070000         1.5290010000
H        -9.5570450000         1.7150560000        -0.1875720000
H         9.5557910000        -1.7149170000        -0.1876660000
C         0.6725990000        -0.2746890000        -0.1644910000
C         1.0702790000        -1.5819100000        -0.3229410000
C         2.4810330000        -1.7438510000        -0.2903670000
C         3.1626520000        -0.5628790000        -0.1082090000
S         2.0482430000         0.7587250000         0.0506140000
C         4.6111180000        -0.3425160000        -0.0299770000
C         5.1950230000         0.8912260000        -0.3497730000
C         6.5669060000         1.0906310000        -0.2881120000
C         7.4371460000         0.0466570000         0.0973170000
C         6.8492870000        -1.1974510000         0.4206910000
C         5.4750970000        -1.3755380000         0.3614490000
N         8.7987290000         0.2337270000         0.1579160000
H         9.4428760000        -1.2469300000         1.5288850000
H        10.7009460000        -0.5357770000         0.4939220000
H        -2.9839920000         2.6990780000        -0.4332710000
H        -0.3628520000         2.3952300000        -0.4851350000
H        -5.0582240000         2.3437260000         0.6451300000
H        -7.4688450000         2.0356320000         0.7340120000
H        -6.9632590000        -2.0692560000        -0.5519690000
H        -4.5592940000        -1.7194010000        -0.6719170000
H         0.3630840000        -2.3956170000        -0.4859810000
H         2.9843060000        -2.6992570000        -0.4340540000
H         4.5591260000         1.7187070000        -0.6735790000
H         6.9630950000         2.0689070000        -0.5537700000
H         7.4689710000        -2.0347800000         0.7358970000
H         5.0583240000        -2.3432380000         0.6469330000
```

Table S3. Optimized geometry S_0 of DMA-2T (M06-2X/SVP, gas phase).

B.4. OPTIMIZED GEOMETRY S_1 OF DMA-2T

```
52
XYZ file generated by gabedit : coordinates in Angstrom
C          3.1607660000         -0.5391190000         -0.0335040000
C          2.4486410000         -1.7543130000         -0.0398700000
C          1.0708970000         -1.6083870000         -0.0456640000
C          0.6505660000         -0.2543290000         -0.0445670000
S          2.0476180000          0.8149040000         -0.0379190000
C          4.5791360000         -0.3388980000         -0.0193840000
C          5.4821970000         -1.4316510000         -0.0215330000
C          6.8502790000         -1.2536490000         -0.0008580000
C          7.4234410000          0.0444080000          0.0244040000
C          6.5295500000          1.1435050000          0.0227280000
C          5.1609340000          0.9531060000          0.0011620000
N          8.7836700000          0.2259280000          0.0505110000
C          9.3292080000          1.5614250000          0.0838540000
H         10.4229750000          1.5049370000          0.1065810000
H          9.0362100000          2.1471140000         -0.8046000000
H          8.9970800000          2.1180070000          0.9773170000
C         -9.3293060000         -1.5612170000          0.0842410000
H        -10.4230750000         -1.5046620000          0.1066790000
H         -9.0361190000         -2.1474590000         -0.8037880000
H         -8.9974190000         -2.1172640000          0.9781240000
C          9.6615700000         -0.9192450000          0.0546140000
C         -9.6615350000          0.9194550000          0.0532820000
H         10.7019360000         -0.5775290000          0.0848100000
H          9.4883570000         -1.5652780000          0.9325850000
H          9.5319770000         -1.5389690000         -0.8495060000
H         -9.5300690000          1.5395340000         -0.8503010000
C         -0.6505570000          0.2541520000         -0.0444500000
C         -1.0708480000          1.6082230000         -0.0451680000
C         -2.4485870000          1.7541900000         -0.0392210000
C         -3.1607450000          0.5390150000         -0.0330990000
S         -2.0476390000         -0.8150410000         -0.0379600000
C         -4.5791240000          0.3388510000         -0.0189440000
C         -5.1609830000         -0.9531250000          0.0017070000
C         -6.5296110000         -1.1434570000          0.0231390000
C         -7.4234520000         -0.0443190000          0.0244940000
C         -6.8502230000          1.2537110000         -0.0006030000
C         -5.4821300000          1.4316480000         -0.0211420000
N         -8.7836990000         -0.2257680000          0.0502050000
H         -9.4900280000          1.5651410000          0.9318730000
H        -10.7019850000          0.5778000000          0.0812320000
H          2.9473580000         -2.7222840000         -0.0379110000
H          0.3607370000         -2.4353100000         -0.0499360000
H          5.0941180000         -2.4506350000         -0.0399400000
H          7.4875110000         -2.1361870000         -0.0037750000
H          6.9123160000          2.1625400000          0.0395020000
H          4.5133250000          1.8327700000          0.0023240000
H         -0.3606660000          2.4351280000         -0.0492750000
H         -2.9472680000          2.7221780000         -0.0369290000
H         -4.5134090000         -1.8328140000          0.0030870000
H         -6.9124310000         -2.1624700000          0.0400500000
H         -7.4874080000          2.1362820000         -0.0033790000
H         -5.0940060000          2.4506140000         -0.0394670000
```

Table S4. Optimized geometry S_1 of DMA-2T (M06-2X/SVP, gas phase).

B.5. OPTIMIZED GEOMETRY S_0 OF DMA-3T

```
59
XYZ file generated by gabedit : coordinates in Angstrom
C          5.1487680000        -0.6232670000        -0.1575280000
C          4.6247620000        -1.8851920000        -0.3193100000
C          3.2087850000        -1.8983660000        -0.4301380000
C          2.6492640000        -0.6440650000        -0.3531840000
S          3.8779600000         0.5579760000        -0.1193980000
C          6.5519320000        -0.2235890000        -0.0030070000
C          7.5123380000        -1.1312350000         0.4672970000
C          8.8444730000        -0.7750470000         0.6159110000
C          9.2893830000         0.5300870000         0.3036230000
C          8.3208060000         1.4475250000        -0.1616920000
C          6.9940240000         1.0706670000        -0.3112900000
N         10.6081110000         0.8918940000         0.4496240000
C         11.5640030000        -0.0677560000         0.9452080000
H         11.6253170000        -0.9567810000         0.2945780000
H         12.5567790000         0.3946760000         0.9778290000
H         11.3156470000        -0.4133730000         1.9641910000
C         11.0142780000         2.2448590000         0.1573150000
H         10.4946140000         2.9801430000         0.7963790000
H         12.0911320000         2.3452980000         0.3323880000
H         10.8148490000         2.5139520000        -0.8941500000
C          1.2504810000        -0.2618930000        -0.4289570000
C          0.7095060000         0.9786350000        -0.6784460000
C         -0.7095030000         0.9786360000        -0.6784550000
C         -1.2504820000        -0.2618910000        -0.4289730000
S         -0.0000030000        -1.4369230000        -0.1693950000
C         -6.5519340000        -0.2235890000        -0.0030300000
C         -7.5123340000        -1.1312380000         0.4672780000
C         -8.8444680000        -0.7750510000         0.6159050000
C         -9.2893820000         0.5300830000         0.3036260000
C         -8.3208100000         1.4475250000        -0.1616900000
C         -6.9940290000         1.0706700000        -0.3113010000
N        -10.6081100000         0.8918890000         0.4496340000
C        -11.0142720000         2.2448630000         0.1573600000
C        -11.5639950000        -0.0677550000         0.9452450000
H        -11.6253130000        -0.9567900000         0.2946300000
H        -11.3156300000        -0.4133550000         1.9642320000
H        -10.4946090000         2.9801300000         0.7964460000
H        -12.0911260000         2.3453000000         0.3324280000
H        -10.8148360000         2.5139810000        -0.8940960000
H        -12.5567720000         0.3946760000         0.9778640000
C         -5.1487710000        -0.6232640000        -0.1575670000
C         -4.6247670000        -1.8851860000        -0.3193890000
C         -3.2087900000        -1.8983590000        -0.4302150000
C         -2.6492660000        -0.6440620000        -0.3532170000
S         -3.8779610000         0.5579760000        -0.1194070000
H          5.2455710000        -2.7776760000        -0.3878370000
H          2.6151700000        -2.7992040000        -0.5884150000
H          7.2052700000        -2.1405720000         0.7474000000
H          9.5426270000        -1.5194550000         0.9943210000
H          8.6034840000         2.4662620000        -0.4199170000
H          6.2813740000         1.8052330000        -0.6942550000
H          1.3223620000         1.8577570000        -0.8784690000
H         -1.3223560000         1.8577570000        -0.8784860000
H         -7.2052610000        -2.1405750000         0.7473750000
H         -9.5426180000        -1.5194620000         0.9943190000
H         -8.6034910000         2.4662640000        -0.4199040000
H         -6.2813820000         1.8052370000        -0.6942660000
H         -5.2455800000        -2.7776650000        -0.3879450000
H         -2.6151770000        -2.7991930000        -0.5885200000
```

Table S5. Optimized geometry S_0 of DMA-3T (M06-2X/SVP, gas phase).

B.6. OPTIMIZED GEOMETRY S_1 OF DMA-3T

```
59
XYZ file generated by gabedit : coordinates in Angstrom
C          5.1887630000         -0.5959940000         -0.0001360000
C          4.6157950000         -1.8759290000         -0.0001350000
C          3.2183020000         -1.8880440000         -0.0001900000
C          2.6386610000         -0.6022510000         -0.0002420000
S          3.9121620000          0.6223160000         -0.0002140000
C          6.5836800000         -0.2255970000         -0.0000650000
C          7.6155750000         -1.2006880000          0.0001980000
C          8.9576240000         -0.8578100000          0.0003130000
C          9.3746510000          0.5015940000          0.0001870000
C          8.3493330000          1.4841610000         -0.0001320000
C          7.0096350000          1.1276640000         -0.0002430000
N         10.7110200000          0.8490000000          0.0003410000
C         11.7309430000         -0.1793220000          0.0007860000
H         11.6670460000         -0.8306550000         -0.8907750000
H         12.7227710000          0.2902200000          0.0011390000
H         11.6663480000         -0.8305330000          0.8923800000
C         11.0987730000          2.2447050000          0.0002330000
H         10.7234540000          2.7813440000          0.8914610000
H         12.1935390000          2.3195640000          0.0005520000
H         10.7239950000          2.7810680000         -0.8913920000
C          1.2728350000         -0.2410350000         -0.0002770000
C          0.6952440000          1.0493680000         -0.0003270000
C         -0.6952440000          1.0493680000         -0.0003240000
C         -1.2728350000         -0.2410350000         -0.0002700000
S          0.0000000000         -1.4719140000         -0.0002240000
C         -6.5836800000         -0.2255960000         -0.0000560000
C         -7.6155750000         -1.2006870000          0.0002130000
C         -8.9576240000         -0.8578100000          0.0003230000
C         -9.3746510000          0.5015950000          0.0001850000
C         -8.3493330000          1.4841610000         -0.0001410000
C         -7.0096350000          1.1276650000         -0.0002470000
N        -10.7110200000          0.8490000000          0.0003350000
C        -11.0987750000          2.2447040000          0.0002040000
C        -11.7309420000         -0.1793240000          0.0007780000
H        -11.6670470000         -0.8306540000         -0.8907850000
H        -11.6663440000         -0.8305370000          0.8923700000
H        -10.7234530000          2.7813580000          0.8914210000
H        -12.1935410000          2.3195620000          0.0005260000
H        -10.7240010000          2.7810530000         -0.8914310000
H        -12.7227710000          0.2902180000          0.0011370000
C         -5.1887630000         -0.5959940000         -0.0001220000
C         -4.6157950000         -1.8759280000         -0.0001170000
C         -3.2183020000         -1.8880440000         -0.0001740000
C         -2.6386610000         -0.6022510000         -0.0002280000
S         -3.9121620000          0.6223160000         -0.0001860000
H          5.2150940000         -2.7871020000         -0.0001100000
H          2.6174460000         -2.7988140000         -0.0002110000
H          7.3556000000         -2.2609650000          0.0003260000
H          9.6954610000         -1.6595370000          0.0004990000
H          8.6015840000          2.5440320000         -0.0003100000
H          6.2630890000          1.9259050000         -0.0004960000
H          1.3028470000          1.9557930000         -0.0003720000
H         -1.3028470000          1.9557930000         -0.0003660000
H         -7.3556000000         -2.2609650000          0.0003520000
H         -9.6954610000         -1.6595360000          0.0005140000
H         -8.6015840000          2.5440320000         -0.0003300000
H         -6.2630890000          1.9259050000         -0.0005060000
H         -5.2150940000         -2.7871020000         -0.0000950000
H         -2.6174460000         -2.7988140000         -0.0002020000
```

Table S6. Optimized geometry S_1 of DMA-3T (M06-2X/SVP, gas phase).

B.7. OPTIMIZED GEOMETRY S_0 OF BHA-1T

```
73
XYZ file generated by gabedit : coordinates in Angstrom
C         2.6792270000        -0.8307720000        -0.2339580000
C         3.0977490000         0.5013710000        -0.3734910000
C         4.4376640000         0.8515730000        -0.2727200000
C         5.4123510000        -0.1237390000        -0.0162440000
C         5.0023600000        -1.4580100000         0.1295220000
C         3.6617470000        -1.8008980000         0.0192850000
N         6.7719540000         0.2307240000         0.1004020000
C         7.7777480000        -0.6283940000        -0.4007640000
C         7.1363590000         1.4409760000         0.7371330000
C         6.4744530000         1.8597670000         1.8995550000
C         6.8318950000         3.0550390000         2.5171440000
C         7.8627870000         3.8397170000         2.0002390000
C         8.5294740000         3.4189710000         0.8495510000
C         8.1667320000         2.2344580000         0.2143720000
C         8.9598990000        -0.8338890000         0.3241640000
C         9.9522160000        -1.6719860000        -0.1764960000
C         9.7770910000        -2.3306640000        -1.3933890000
C         8.5978810000        -2.1337100000        -2.1120310000
C         7.6075180000        -1.2832110000        -1.6285330000
C         1.2664400000        -1.2114700000        -0.3490710000
C         0.7312870000        -2.4286430000        -0.6998020000
C        -0.6895930000        -2.4365440000        -0.7078710000
C        -1.2432660000        -1.2258270000        -0.3629690000
S         0.0034480000        -0.0730060000        -0.0095380000
C        -2.6622190000        -0.8614310000        -0.2652950000
C        -3.6423290000        -1.8478830000        -0.0724780000
C        -4.9898220000        -1.5219270000        -0.0011630000
C        -5.4073340000        -0.1852490000        -0.0916890000
C        -4.4336750000         0.8086560000        -0.2667820000
C        -3.0899550000         0.4714160000        -0.3639650000
N        -6.7743630000         0.1506900000        -0.0063550000
C        -7.3025170000         1.1889070000        -0.8094420000
C        -7.6241330000        -0.5490670000         0.8823940000
C        -7.1827890000        -0.8911450000         2.1681350000
C        -8.0216340000        -1.5863550000         3.0347520000
C        -9.3146580000        -1.9341570000         2.6438140000
C        -9.7587330000        -1.5855860000         1.3683640000
C        -8.9209260000        -0.9066410000         0.4881160000
C        -8.2335540000         2.0917440000        -0.2775930000
C        -8.7605450000         3.1050020000        -1.0735050000
C        -8.3570250000         3.2465500000        -2.4011500000
C        -7.4241840000         2.3541840000        -2.9294390000
C        -6.9054660000         1.3263750000        -2.1468090000
H         2.3614700000         1.2773330000        -0.5941600000
H         4.7415210000         1.8916720000        -0.3974010000
H         5.7470230000        -2.2257380000         0.3436780000
H         3.3642800000        -2.8401270000         0.1684040000
H         5.6756380000         1.2409330000         2.3109920000
H         6.3062470000         3.3682460000         3.4205940000
H         8.1447300000         4.7719780000         2.4906410000
H         9.3341940000         4.0255510000         0.4310430000
H         8.6811920000         1.9106160000        -0.6916230000
H         9.0932180000        -0.3274210000         1.2812220000
H        10.8670790000        -1.8207860000         0.3992940000
H        10.5537020000        -2.9918530000        -1.7791080000
H         8.4501280000        -2.6367850000        -3.0689980000
H         6.6907290000        -1.1204060000        -2.1971820000
H         1.3444970000        -3.2846270000        -0.9796700000
H        -1.2884520000        -3.3006460000        -0.9935890000
H        -3.3427070000        -2.8920410000         0.0288690000
H        -5.7344710000        -2.3063050000         0.1403520000
H        -4.7409570000         1.8528210000        -0.3373120000
H        -2.3551380000         1.2640550000        -0.5206930000
H        -6.1757100000        -0.6090890000         2.4790170000
H        -7.6631420000        -1.8463040000         4.0320360000
H        -9.9713290000        -2.4722120000         3.3281830000
H       -10.7658220000        -1.8560930000         1.0470290000
H        -9.2650490000        -0.6431910000        -0.5131130000
H        -8.5416450000         1.9878760000         0.7638420000
H        -9.4851940000         3.7992570000        -0.6450730000
H        -8.7664760000         4.0459920000        -3.0194800000
H        -7.1042930000         2.4486070000        -3.9683890000
H        -6.1846730000         0.6218370000        -2.5644830000
```

Table S7. Optimized geometry S_0 of BHA-1T (M06-2X/SVP, gas phase).

B.8. OPTIMIZED GEOMETRY S_1 OF BHA-1T

```
73
XYZ file generated by gabedit : coordinates in Angstrom
C        -2.6362730000         -0.8963700000         -0.1348930000
C        -3.0572630000          0.4672160000         -0.1818550000
C        -4.3892440000          0.8152280000         -0.1521860000
C        -5.3951150000         -0.1744980000         -0.0770680000
C        -4.9982170000         -1.5328840000         -0.0323410000
C        -3.6675920000         -1.8815100000         -0.0599580000
N        -6.7511150000          0.1774520000         -0.0483590000
C        -7.6505720000         -0.5449810000          0.7677270000
C        -7.2186280000          1.2560130000         -0.8322410000
C        -6.7108420000          1.4581790000         -2.1248750000
C        -7.1541990000          2.5320380000         -2.8892290000
C        -8.1148550000          3.4113890000         -2.3875530000
C        -8.6265210000          3.2063010000         -1.1054290000
C        -8.1812780000          2.1431830000         -0.3262960000
C        -8.9398040000         -0.8559320000          0.3098140000
C        -9.8128670000         -1.5739270000          1.1209080000
C        -9.4152880000         -2.0031390000          2.3881900000
C        -8.1315300000         -1.6984600000          2.8418740000
C        -7.2540430000         -0.9707880000          2.0448980000
C        -1.2640020000         -1.2554800000         -0.1527470000
C        -0.6846480000         -2.5488580000         -0.1187480000
C         0.6971710000         -2.5460710000         -0.1279500000
C         1.2706540000         -1.2502170000         -0.1652880000
S         0.0004500000         -0.0361330000         -0.2085680000
C         2.6409620000         -0.8841490000         -0.1451600000
C         3.6780880000         -1.8635570000         -0.0756160000
C         5.0058520000         -1.5071370000         -0.0152230000
C         5.3933340000         -0.1451960000         -0.0253780000
C         4.3823610000          0.8381760000         -0.1170320000
C         3.0535890000          0.4825900000         -0.1738240000
N         6.7418690000          0.2218260000          0.0680530000
C         7.1120980000          1.3525980000          0.8316080000
C         7.7379940000         -0.5486320000         -0.5718640000
C         7.5049160000         -1.0920620000         -1.8444770000
C         8.4856740000         -1.8594180000         -2.4648980000
C         9.7111600000         -2.0886110000         -1.8378270000
C         9.9447940000         -1.5455060000         -0.5736950000
C         8.9674270000         -0.7875080000          0.0620910000
C         8.1182530000          2.2195470000          0.3790320000
C         8.4761870000          3.3286940000          1.1388040000
C         7.8318610000          3.6000260000          2.3467000000
C         6.8257230000          2.7424530000          2.7933190000
C         6.4685300000          1.6229580000          2.0488370000
H        -2.3061750000          1.2583650000         -0.2267040000
H        -4.6778560000          1.8672220000         -0.1759650000
H        -5.7642140000         -2.3084080000          0.0164840000
H        -3.4007580000         -2.9383790000         -0.0354530000
H        -5.9675680000          0.7636350000         -2.5189070000
H        -6.7511120000          2.6756300000         -3.8930370000
H        -8.4605930000          4.2512970000         -2.9910660000
H        -9.3722660000          3.8915160000         -0.6990440000
H        -8.5711480000          1.9929720000          0.6815720000
H        -9.2467550000         -0.5324690000         -0.6858800000
H       -10.8117800000         -1.8121500000          0.7519480000
H       -10.1009200000         -2.5722110000          3.0169200000
H        -7.8104530000         -2.0213010000          3.8335170000
H        -6.2532040000         -0.7225640000          2.4011840000
H        -1.2832430000         -3.4578500000         -0.0887900000
H         1.2999120000         -3.4525170000         -0.1038080000
H         3.4167150000         -2.9216380000         -0.0448770000
H         5.7742090000         -2.2781350000          0.0603310000
H         4.6658100000          1.8914640000         -0.1465430000
H         2.2997310000          1.2681180000         -0.2552410000
H         6.5508860000         -0.9023480000         -2.3385860000
H         8.2938400000         -2.2728890000         -3.4564100000
H        10.4785350000         -2.6863800000         -2.3307760000
H        10.8956560000         -1.7240370000         -0.0690420000
H         9.1450480000         -0.3746590000          1.0561520000
H         8.6132290000          2.0158010000         -0.5716120000
H         9.2588140000          3.9963080000          0.7749480000
H         8.1113400000          4.4743650000          2.9355840000
H         6.3191430000          2.9386250000          3.7396460000
H         5.6896390000          0.9455990000          2.4017850000
```

Table S8. Optimized geometry S_1 of BHA-1T (M06-2X/SVP, gas phase).

C. MOLECULAR ORBITAL PLOTS OF THE OPTIMIZED S_1 STATE

C.1. DMA-1T

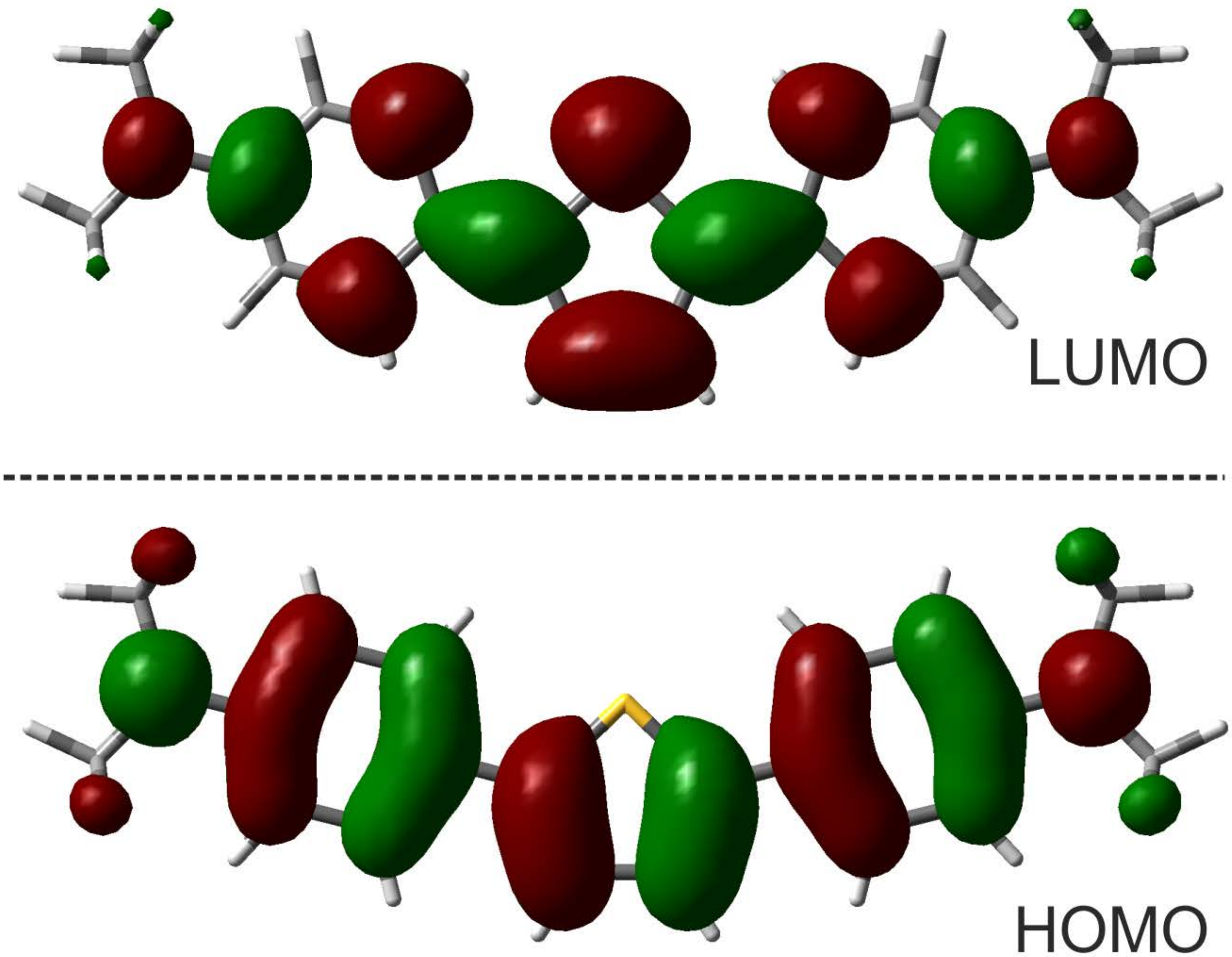

Figure S4. HOMO/LUMO of DMA-1T; B3LYP/SVP, LR-PCM, THF.

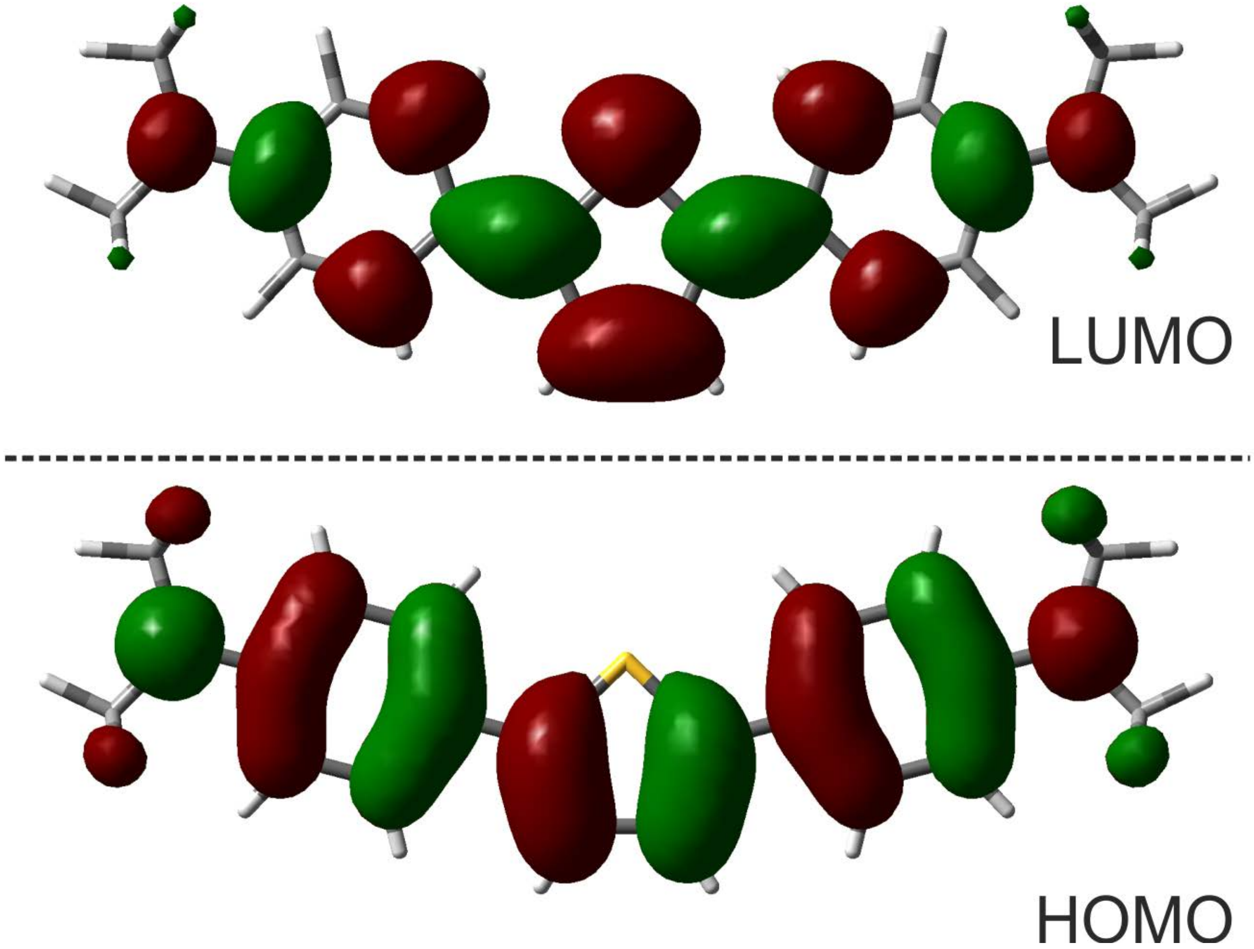

Figure S5. HOMO/LUMO of DMA-1T; PBE0/SVP, LR-PCM, THF.

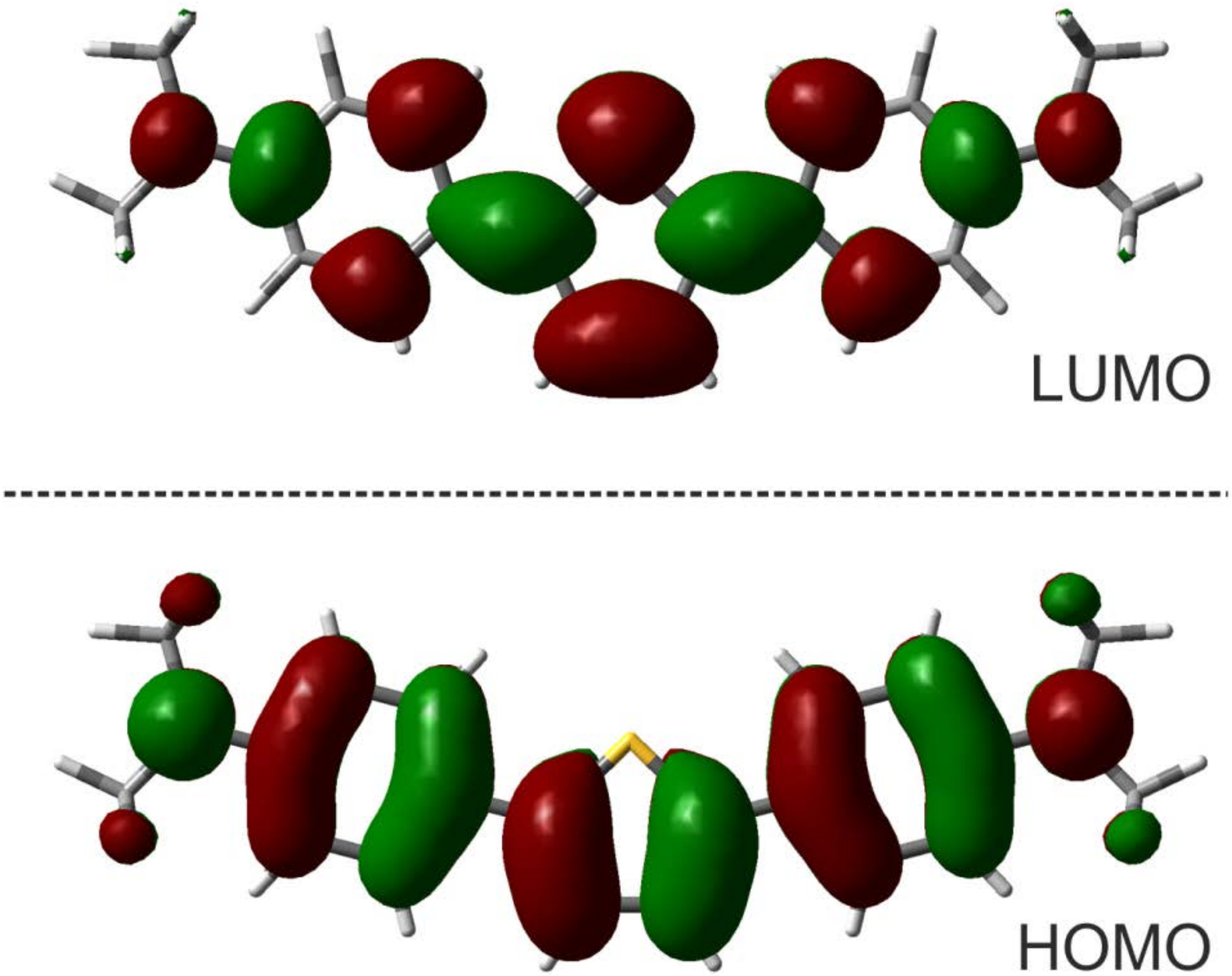

Figure S6. HOMO/LUMO of DMA-1T; M06-2X/SVP, LR-PCM, THF.

C.2. DMA-2T

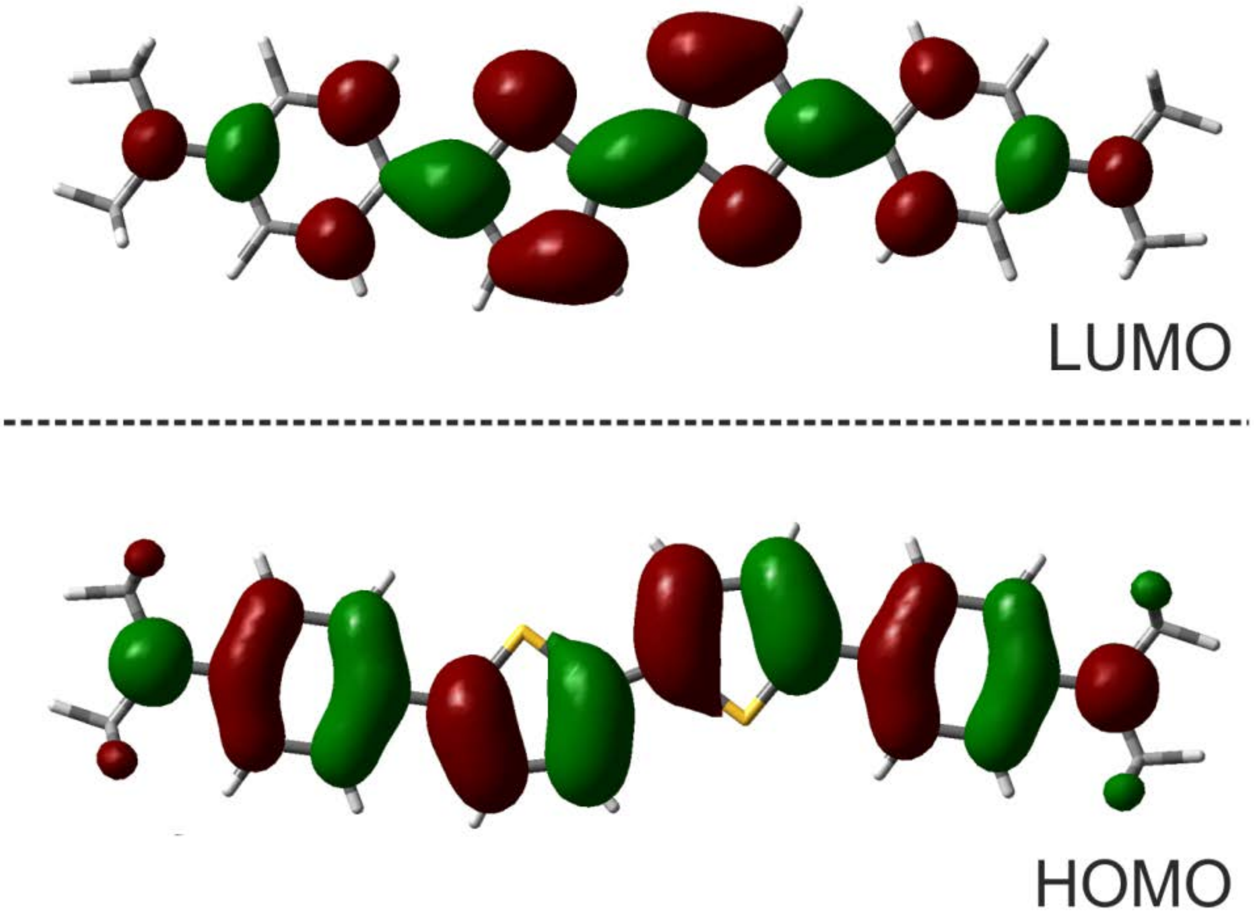

Figure S7. HOMO/LUMO of DMA-2T; B3LYP/SVP, LR-PCM, THF.

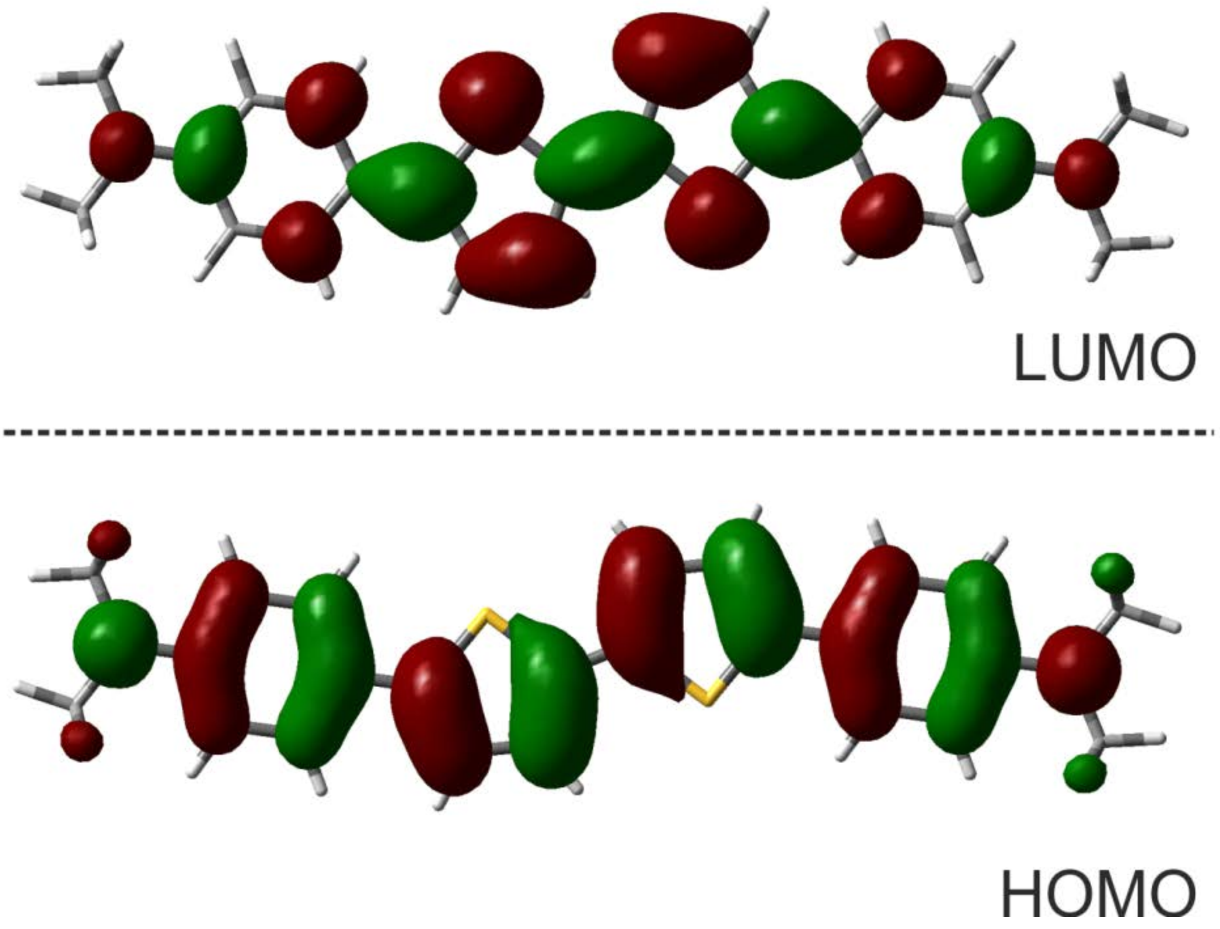

Figure S8. HOMO/LUMO of DMA-2T; PBE0/SVP, LR-PCM, THF.

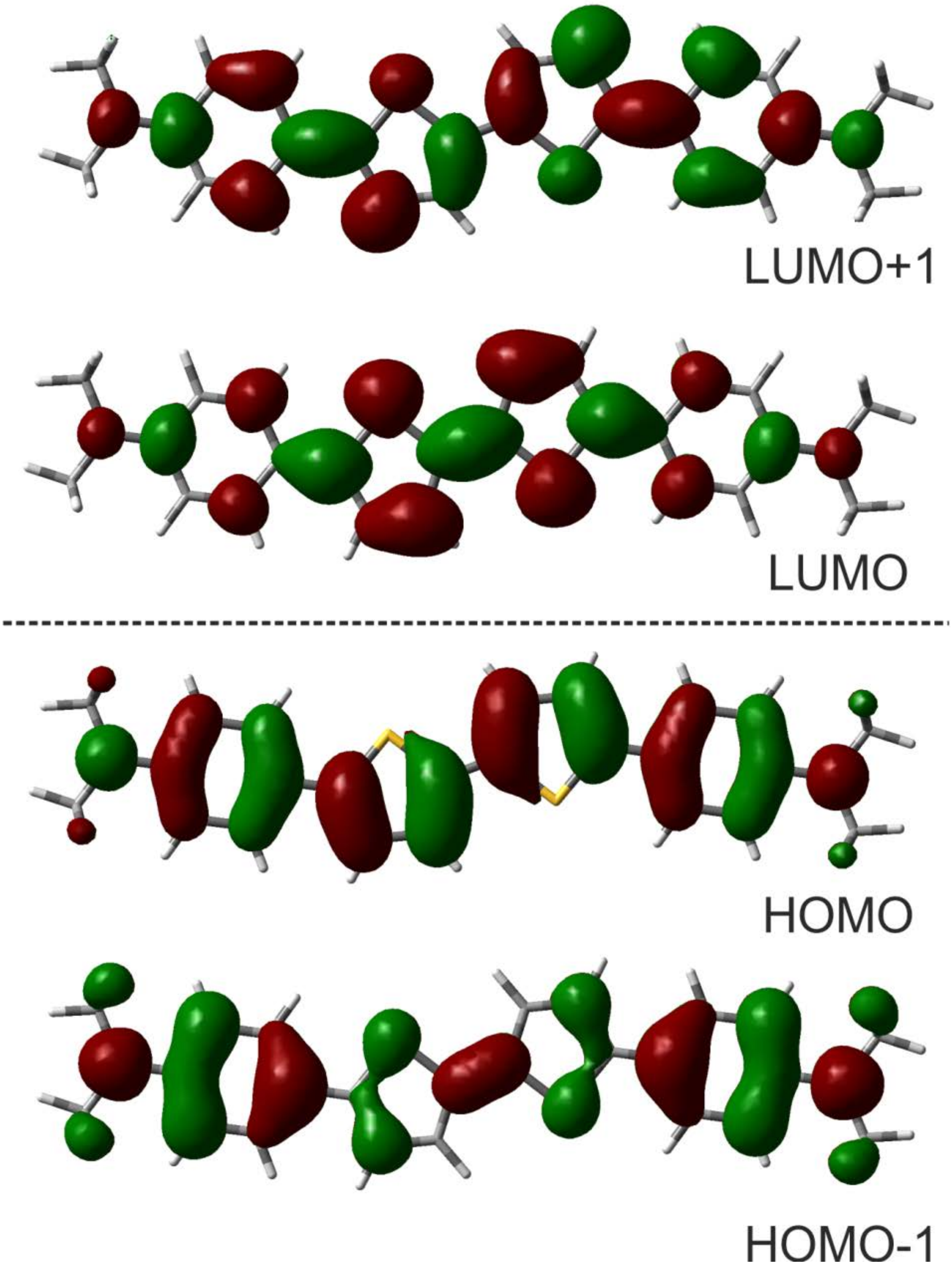

Figure S9. Selected Orbitals of DMA-2T; M06-2X/SVP, LR-PCM, THF.

C.3. DMA-3T

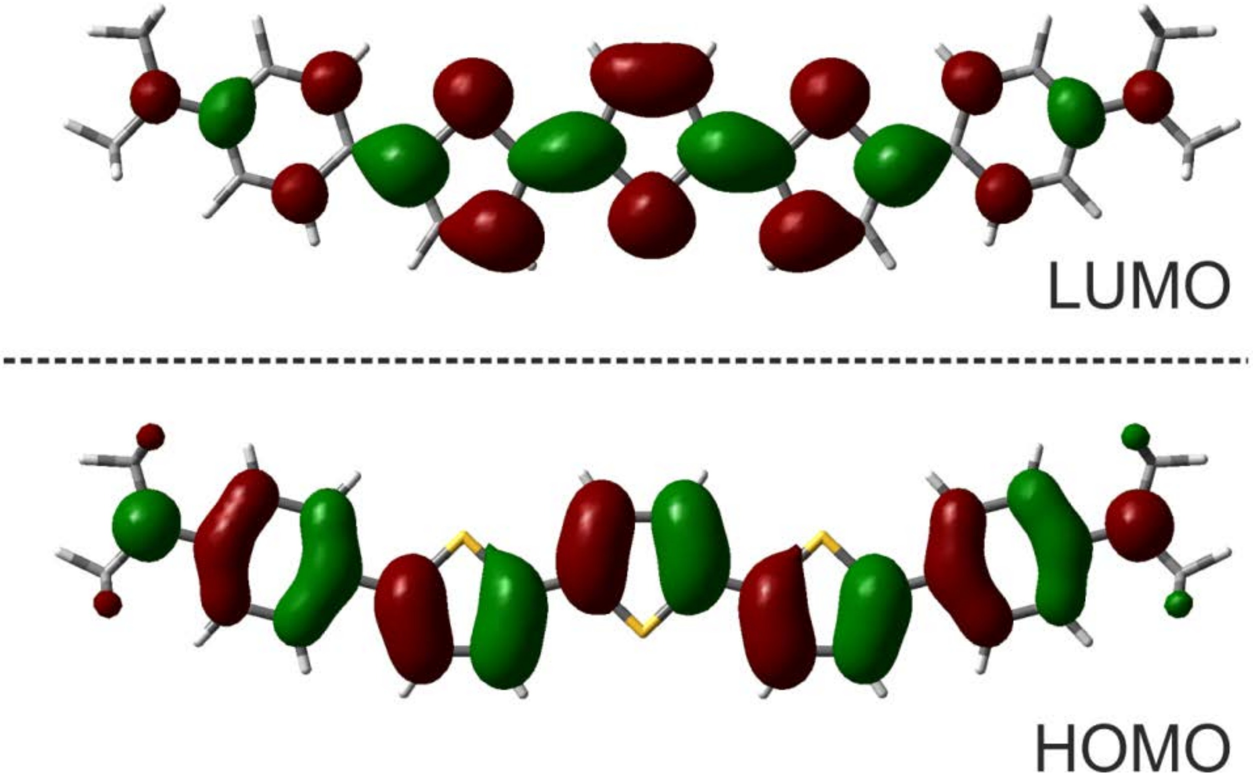

Figure S10. HOMO/LUMO of DMA-3T; B3LYP/SVP, LR-PCM, THF.

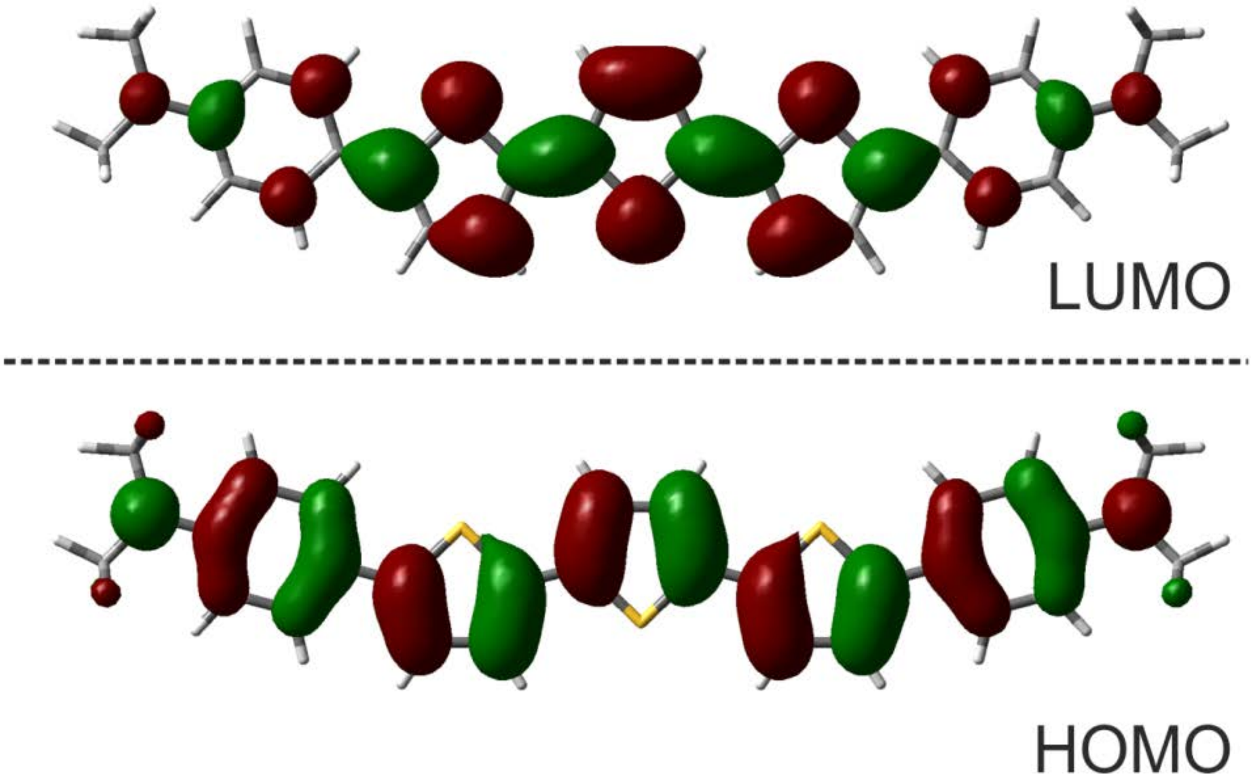

Figure S11. HOMO/LUMO of DMA-3T; PBE0/SVP, LR-PCM, THF.

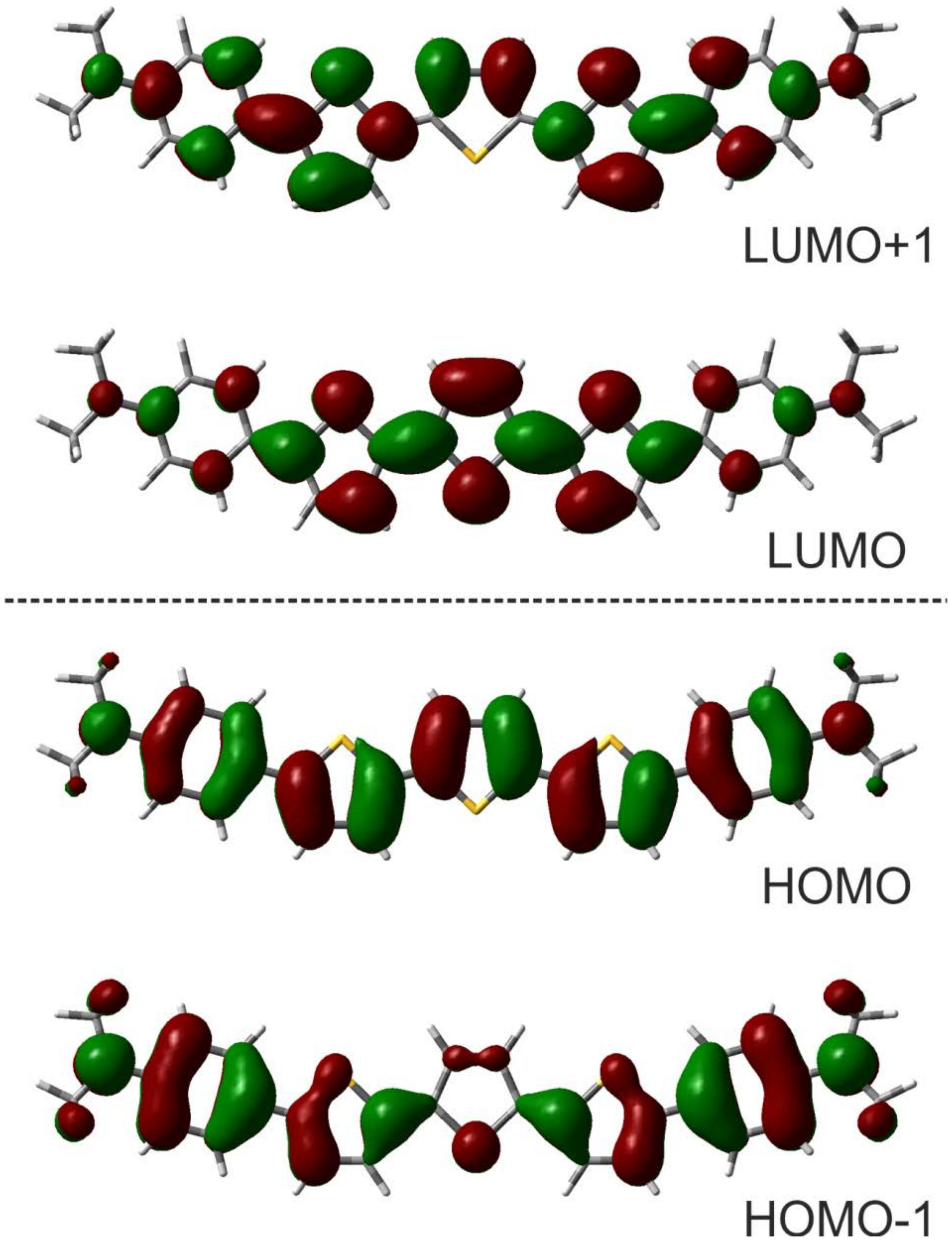

Figure S12. Selected Orbitals of DMA-3T; M06-2X/SVP, LR-PCM, THF.

C.4. BHA-1T

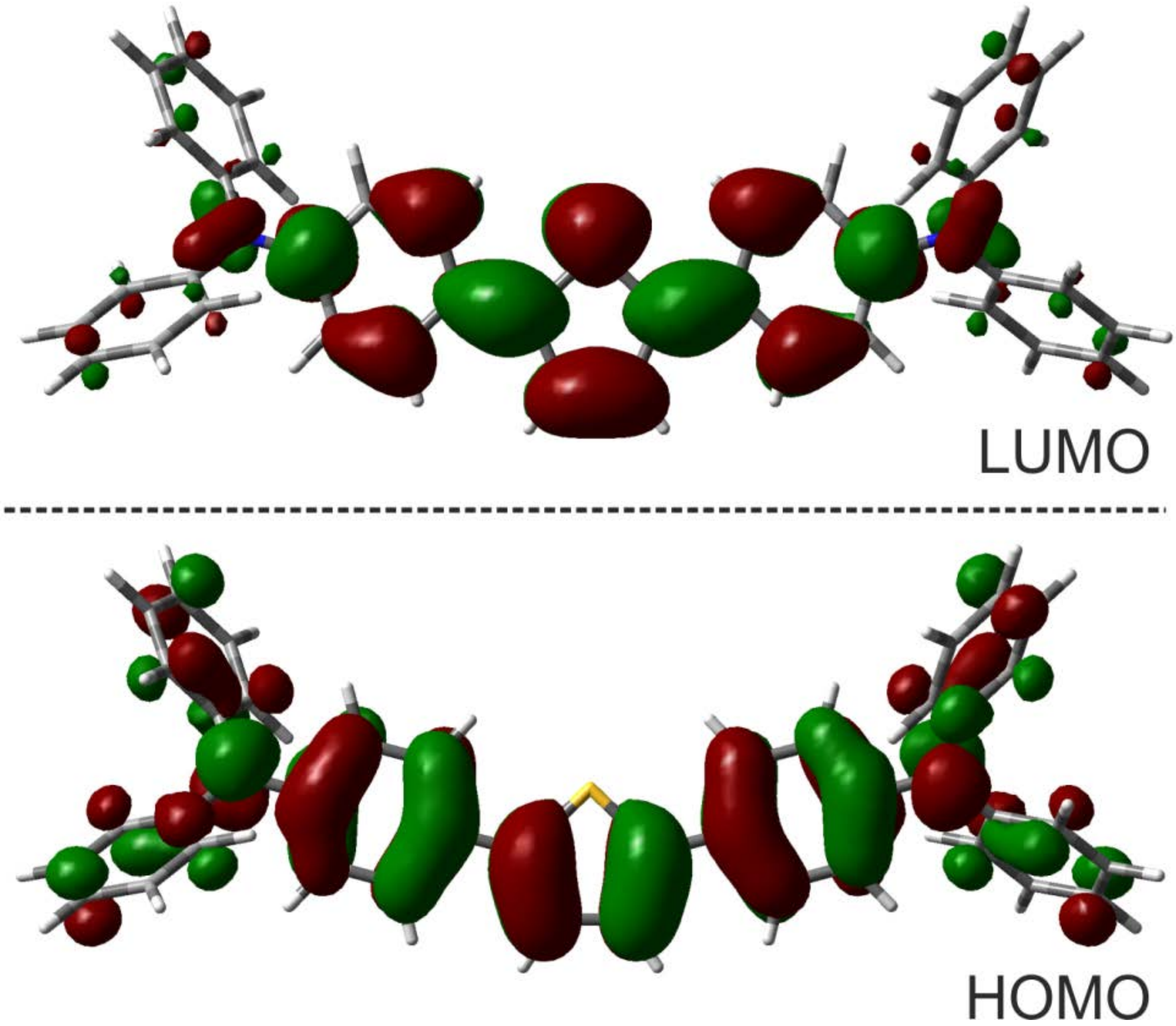

Figure S13. HOMO and LUMO of BHA-1T; M06-2X/SVP, LR-PCM, THF.

D. ABSORPTION AND EMMISSION SPECTRA

D.1. DMA-1T

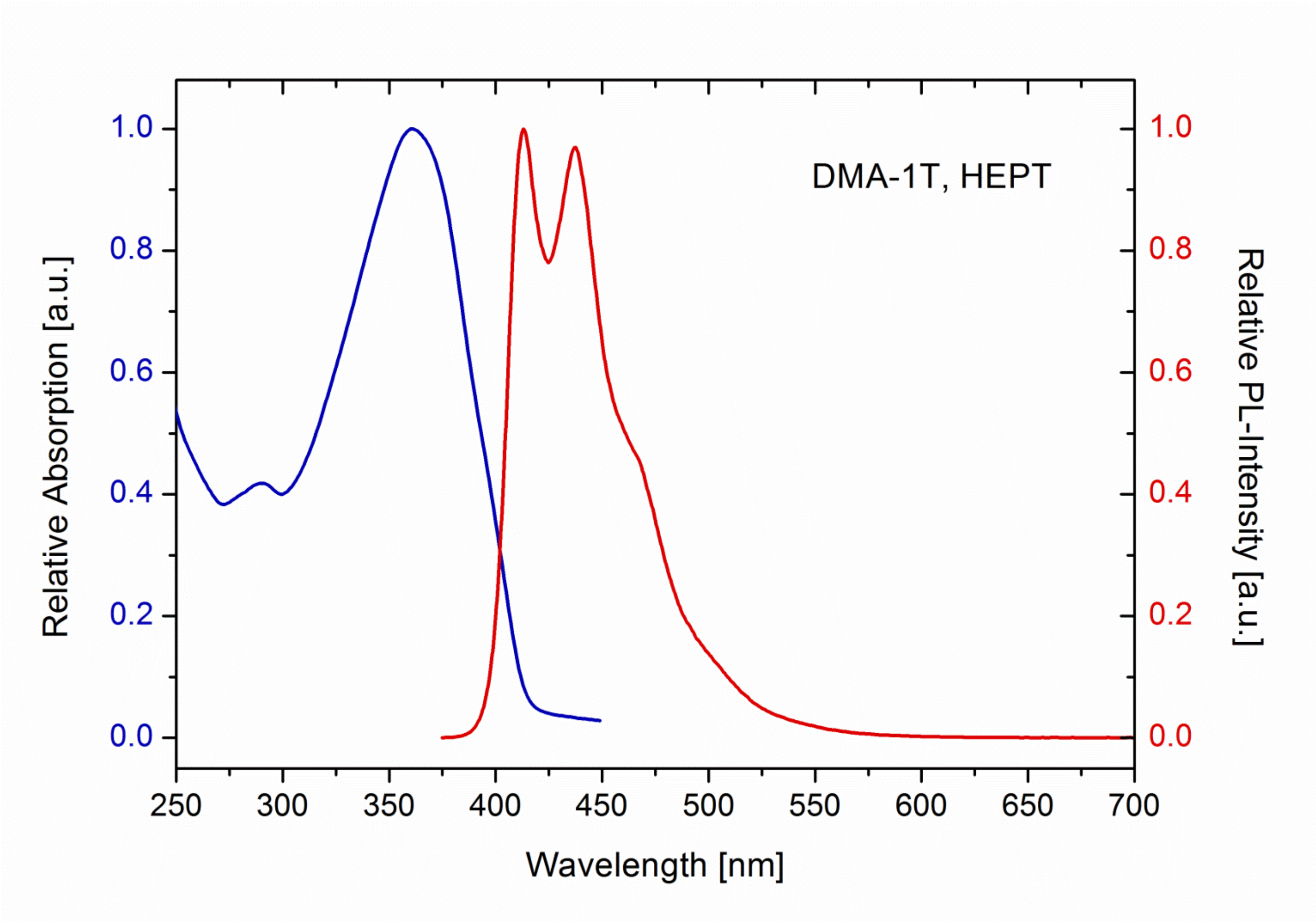

Figure S14. Absorption and emission spectra of DMA-1T, n-heptane.

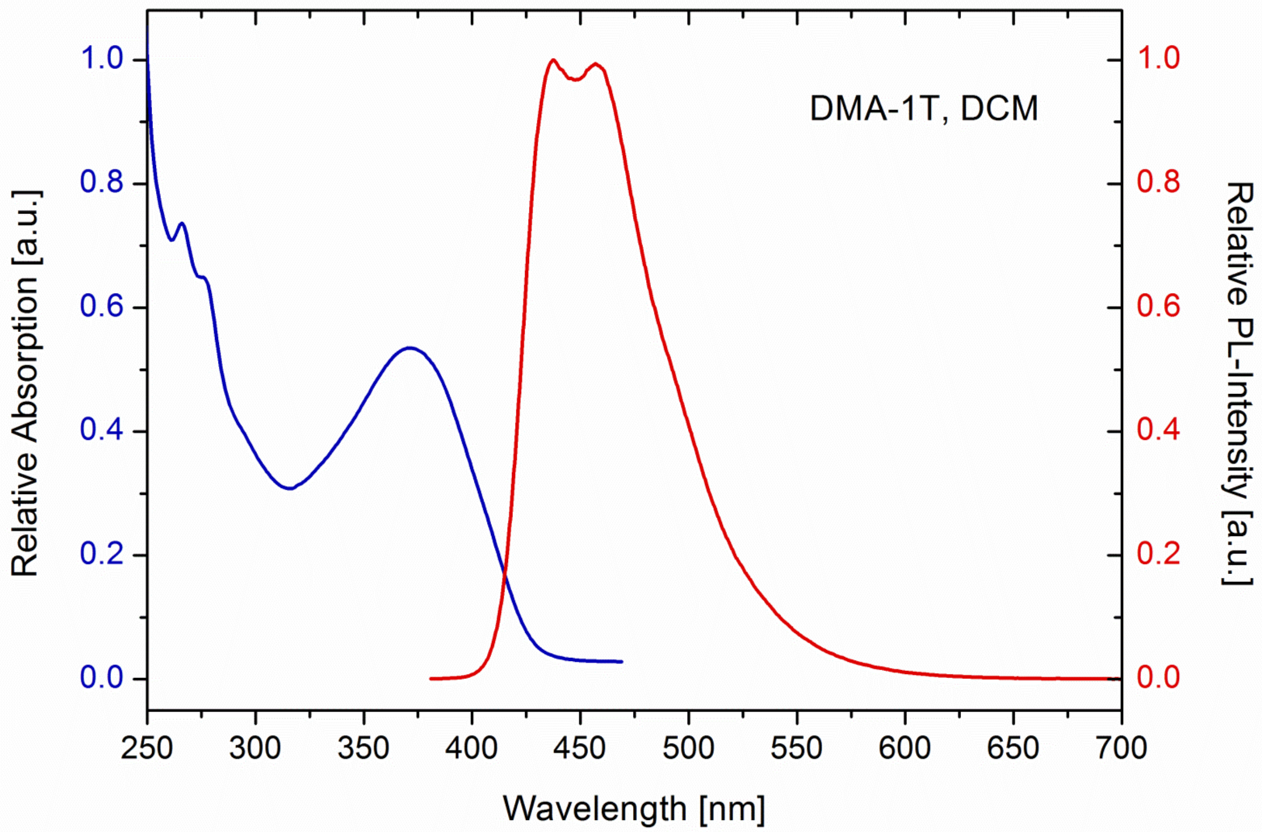

Figure S15. Absorption and emission spectra of DMA-1T, dichloromethane.

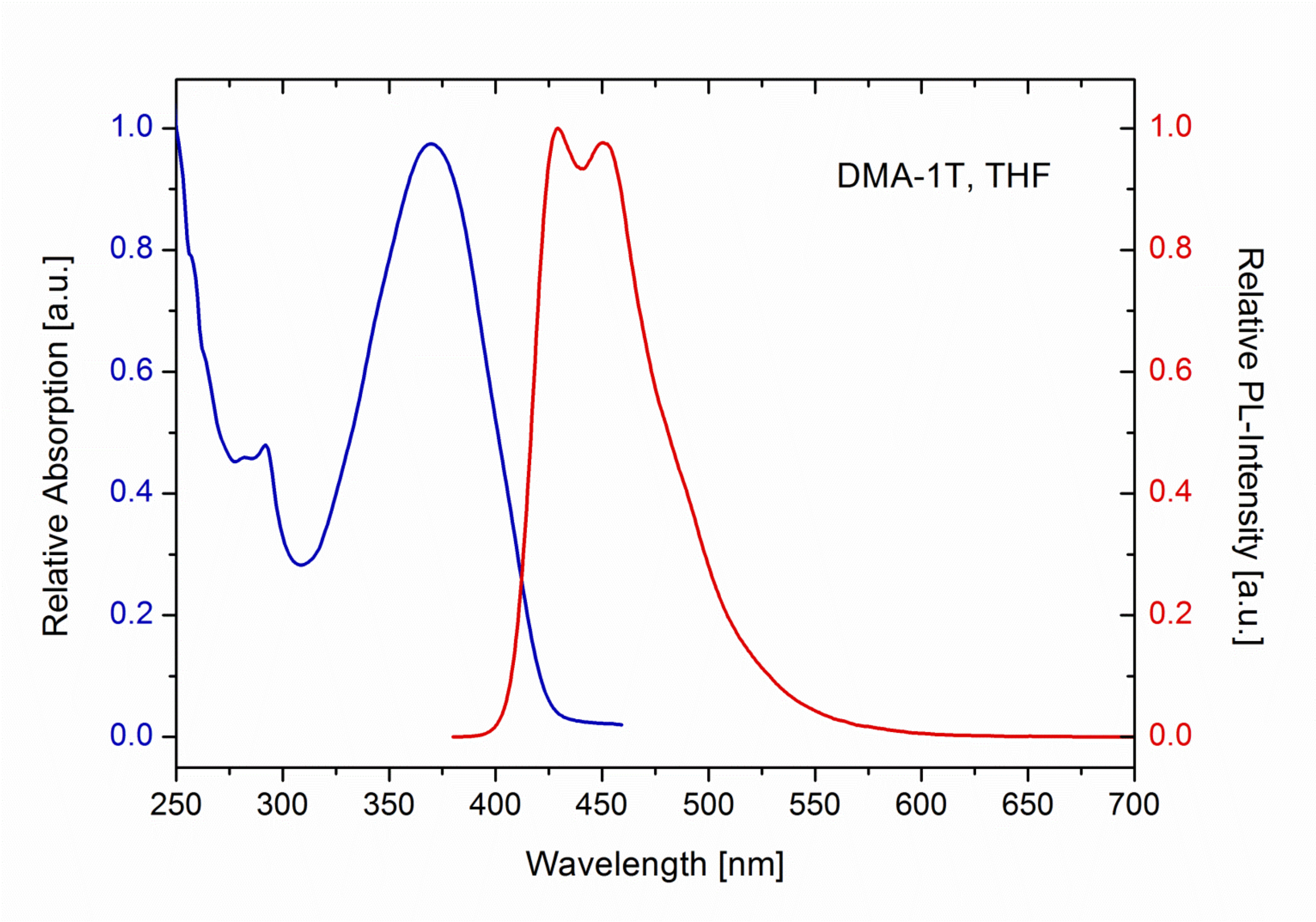

Figure S16. Absorption and emission spectra of DMA-1T, tetrahydrofurane.

D.2. DMA-2T

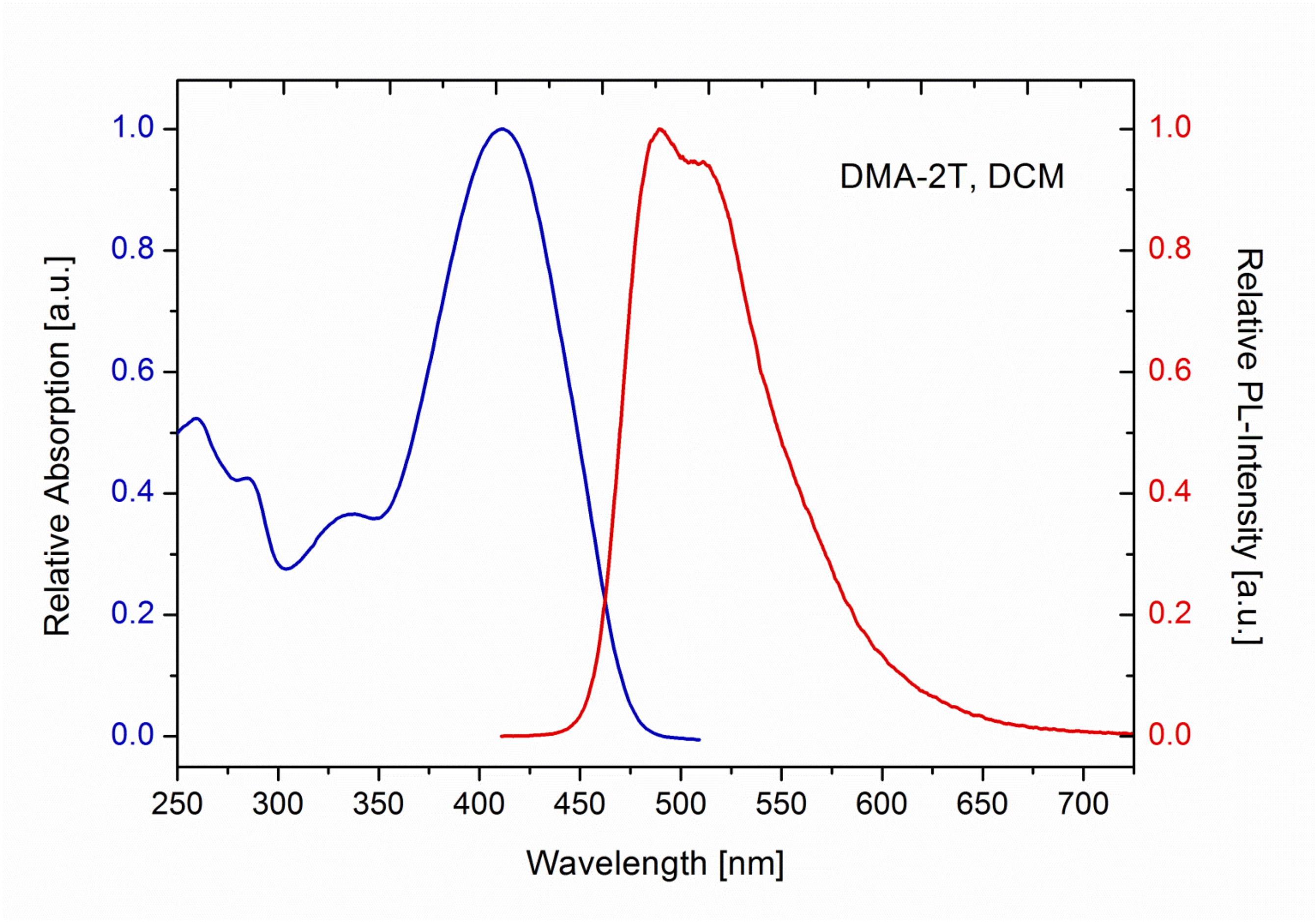

Figure S17. Absorption and emission spectra of DMA-2T, dichloromethane.

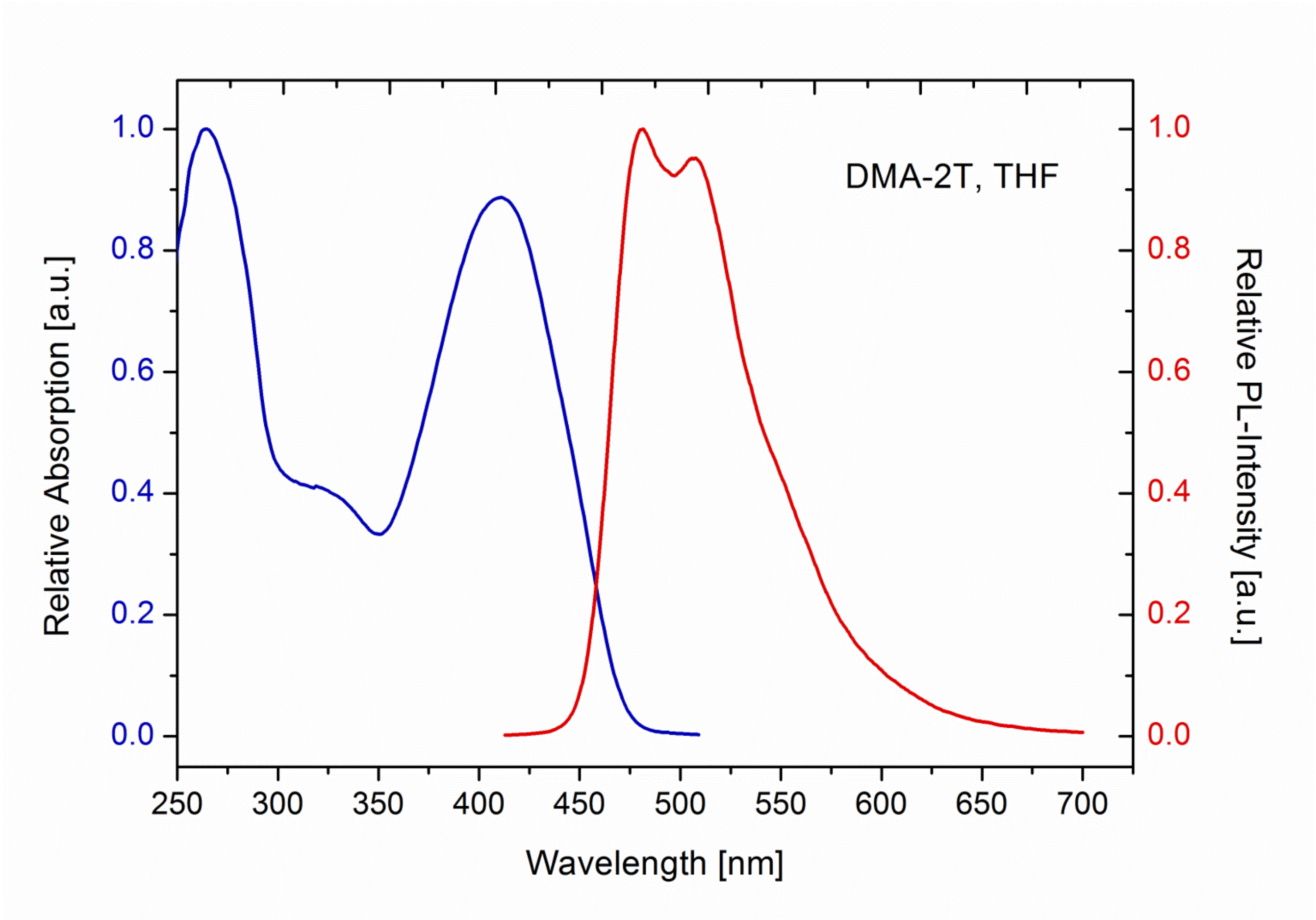

Figure S18. Absorption and emission spectra of DMA-2T, tetrahydrofurane.

D.3. DMA-3T

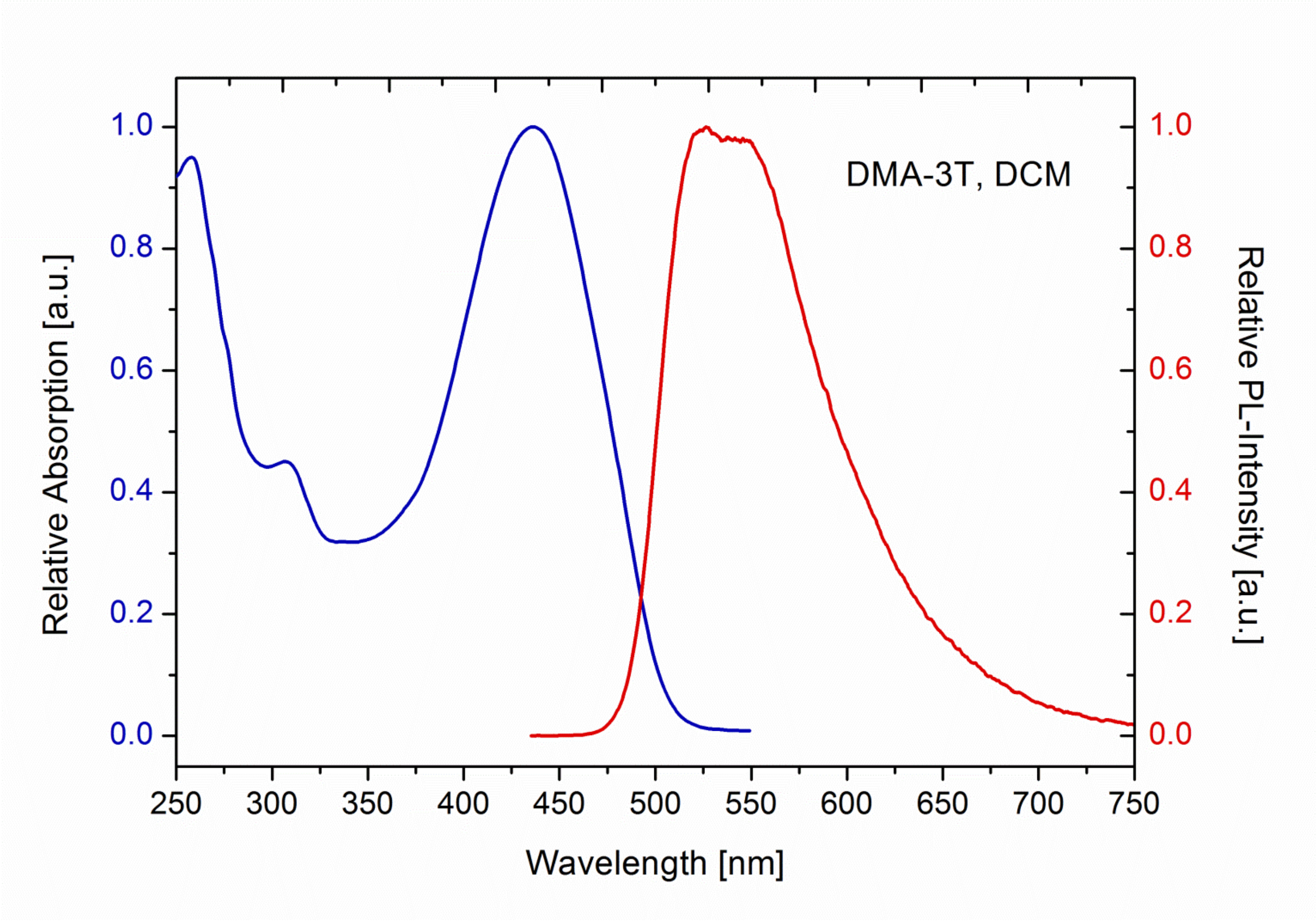

Figure S19. Absorption and emission spectra of DMA-3T, dichloromethane.

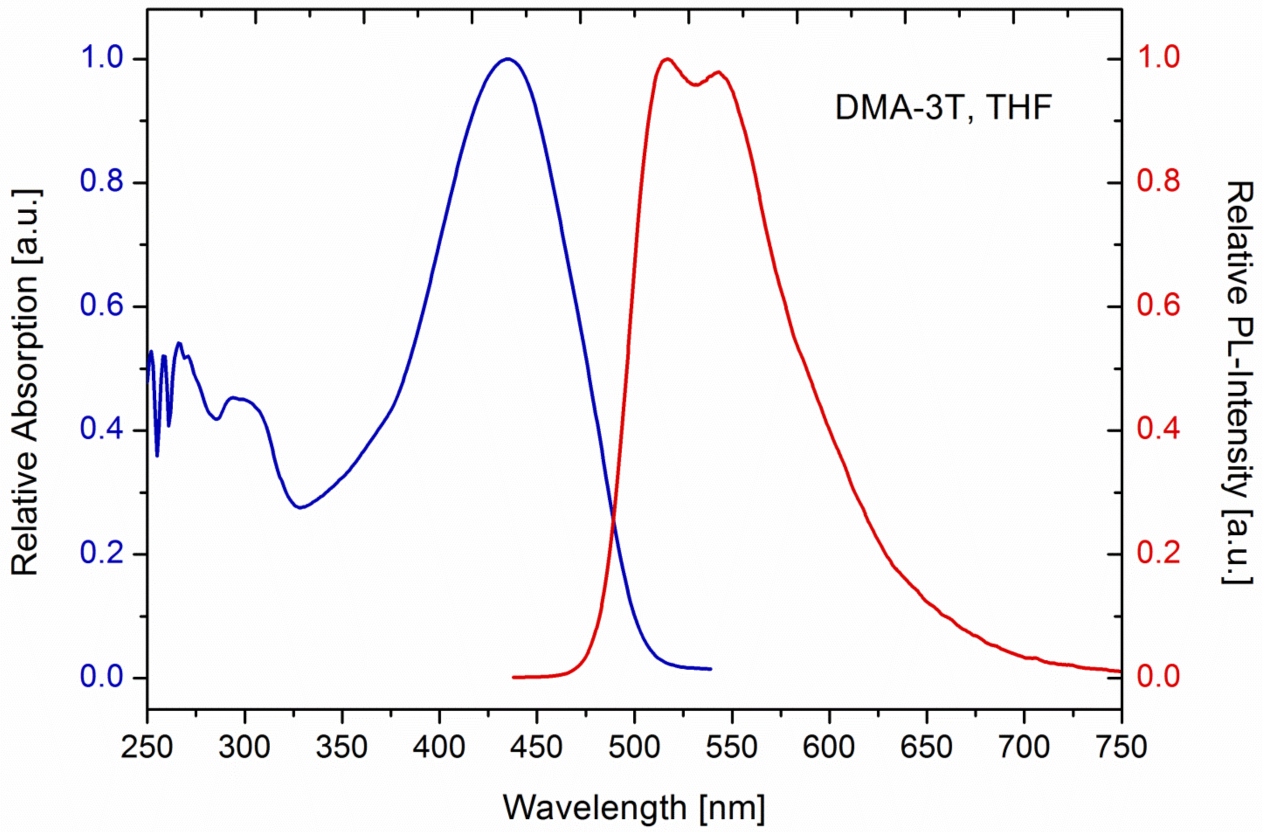

Figure S20. Absorption and emission spectra of DMA-3T, tetrahydrofurane.

D.4. BHA-1T

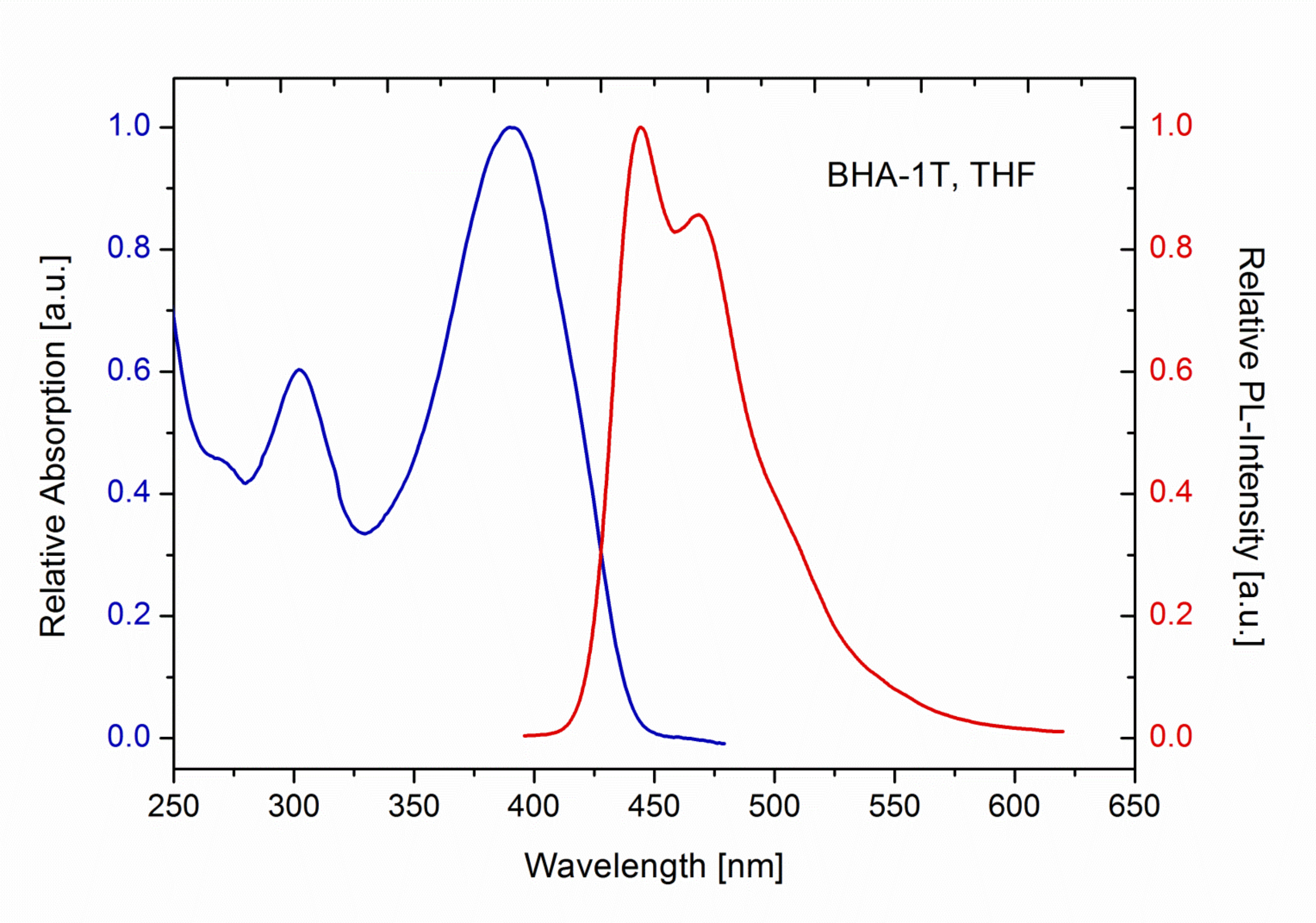

Figure S21. Absorption and emission spectra of BHA-1T, tetrahydrofurane.

E. ^{1}H AND ^{13}C NMR SPECTRA

E.1. DMA-1T

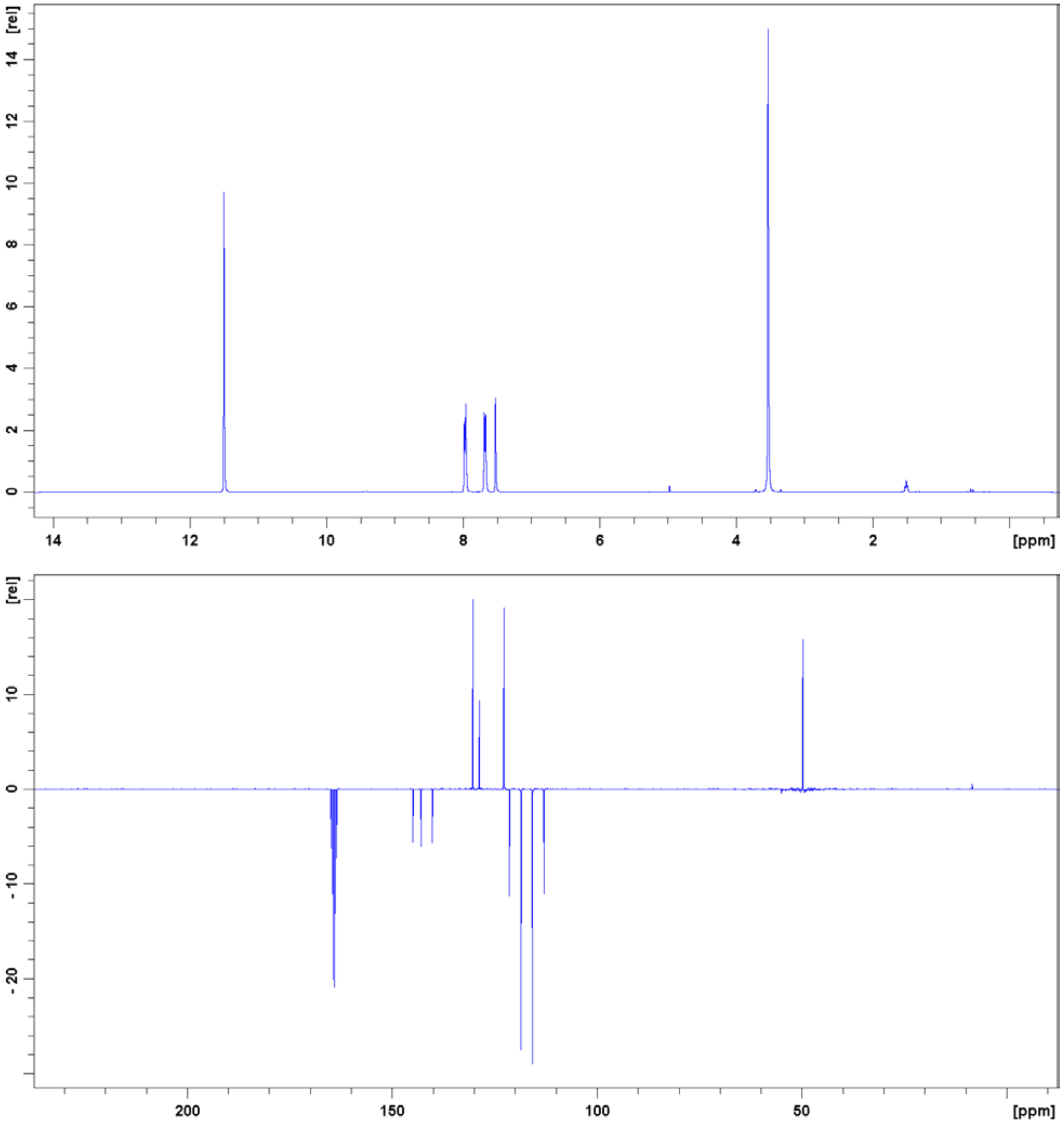

Figure S22. ^{1}H and ^{13}C-APT NMR spectra of DMA-1T, solvent trifluoroacetic acid d1.

E.2. DMA-2T

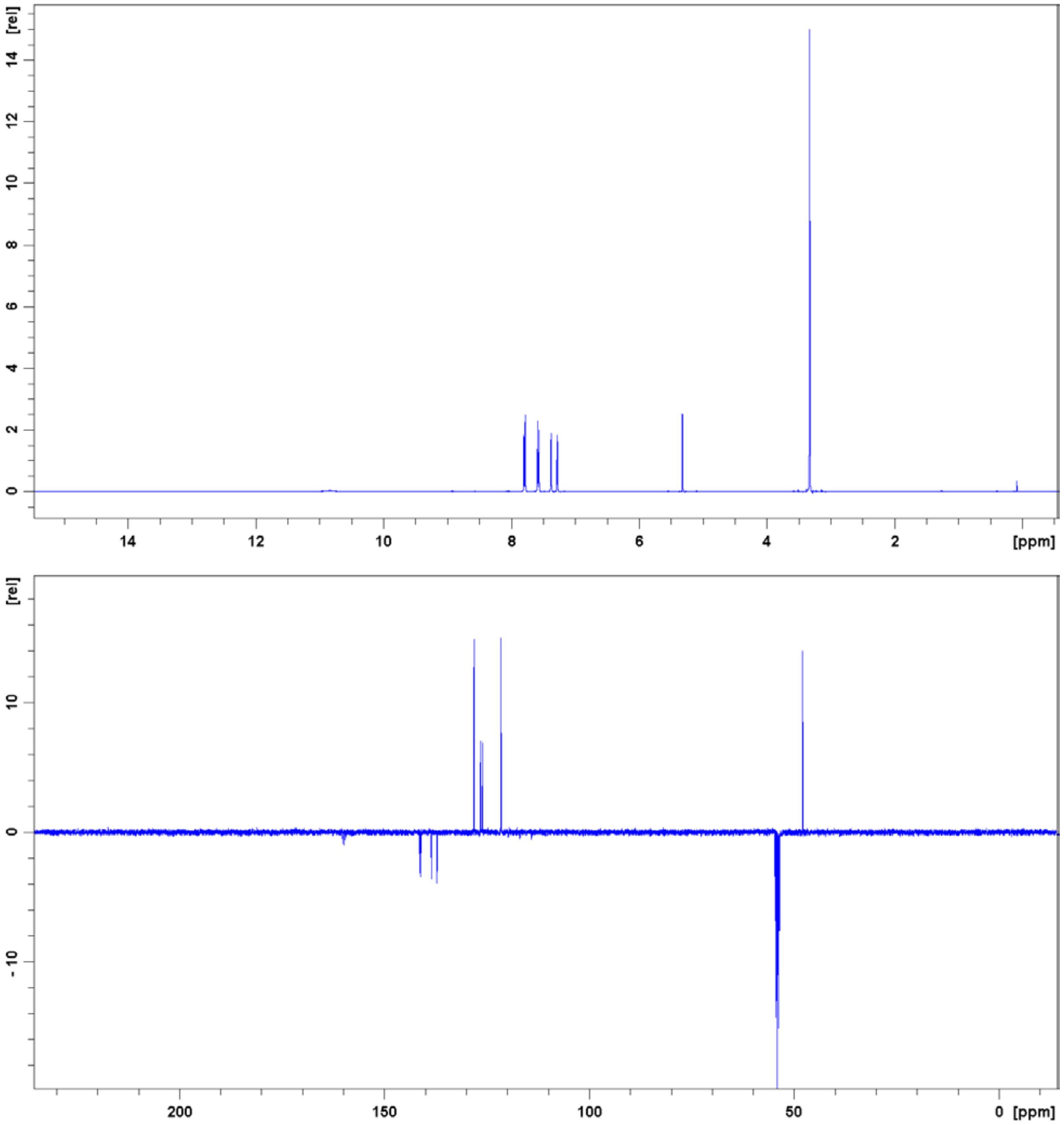

Figure S23. ^{1}H and ^{13}C-APT NMR spectra of DMA-2T, solvent CD_2Cl_2+trifluoroacetic acid d1.

E.3. DMA-3T

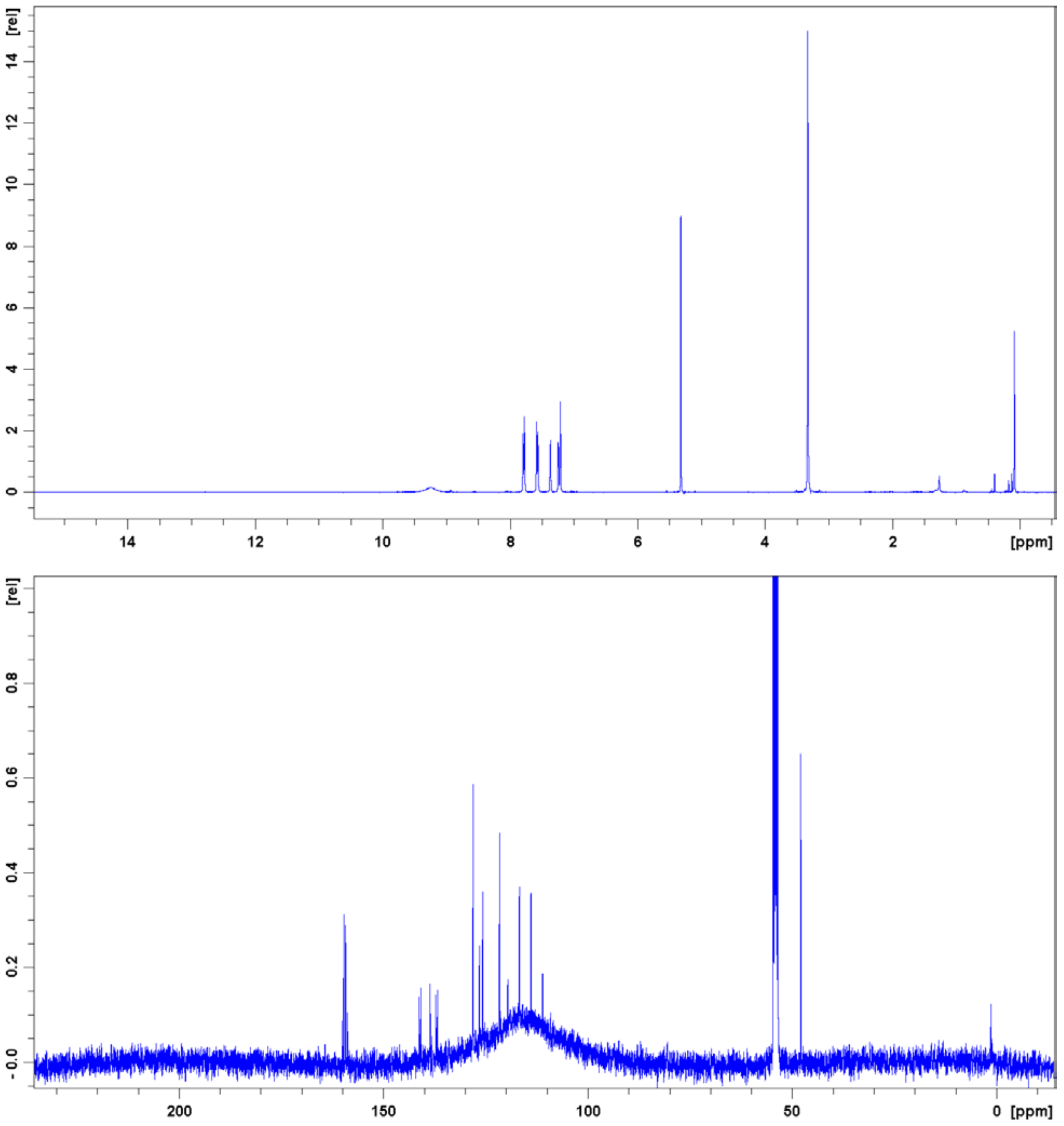

Figure S24. ^{1}H and ^{13}C NMR spectra of DMA-3T, solvent CD_2Cl_2+trifluoroacetic acid d1.

E.4. BHA-1T

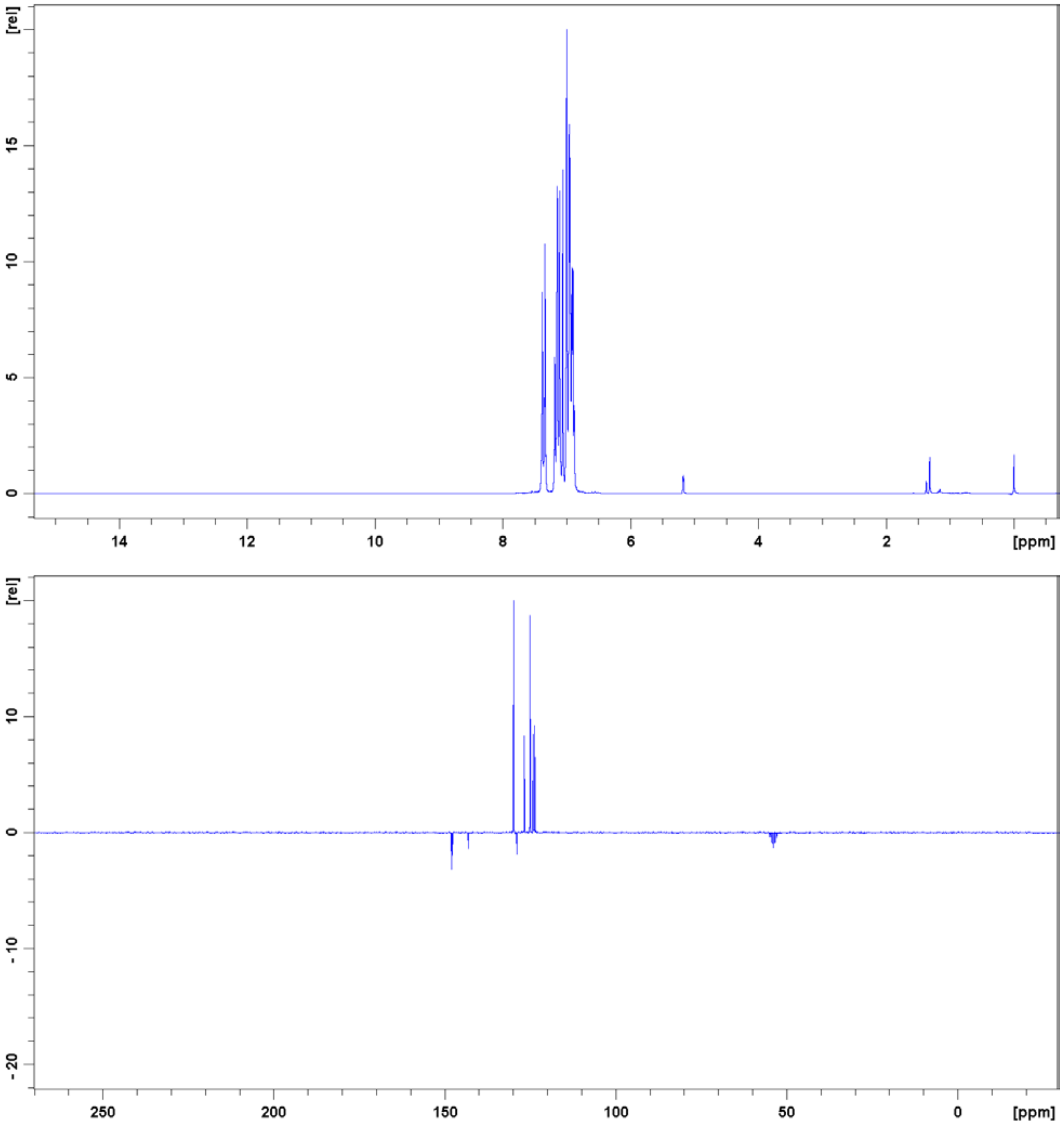

Figure S25. ^{1}H and ^{13}C-APT NMR spectra of BHA-1T, solvent CD_2Cl_2.
